# Electrode replacement does not affect classification accuracy in dual-session use of a passive brain-computer interface for assessing cognitive workload

**DOI:** 10.3389/fnins.2015.00054

**Published:** 2015-03-09

**Authors:** Justin R. Estepp, James C. Christensen

**Affiliations:** Applied Neuroscience Branch, Human Effectiveness Directorate, 711th Human Performance Wing, Air Force Research LaboratoryWright-Patterson AFB, OH, USA

**Keywords:** passive brain computer interface, cognitive state, electroencephalography, machine learning, non-stationarity

## Abstract

The passive brain-computer interface (pBCI) framework has been shown to be a very promising construct for assessing cognitive and affective state in both individuals and teams. There is a growing body of work that focuses on solving the challenges of transitioning pBCI systems from the research laboratory environment to practical, everyday use. An interesting issue is what impact methodological variability may have on the ability to reliably identify (neuro)physiological patterns that are useful for state assessment. This work aimed at quantifying the effects of methodological variability in a pBCI design for detecting changes in cognitive workload. Specific focus was directed toward the effects of replacing electrodes over dual sessions (thus inducing changes in placement, electromechanical properties, and/or impedance between the electrode and skin surface) on the accuracy of several machine learning approaches in a binary classification problem. In investigating these methodological variables, it was determined that the removal and replacement of the electrode suite between sessions does not impact the accuracy of a number of learning approaches when trained on one session and tested on a second. This finding was confirmed by comparing to a control group for which the electrode suite was not replaced between sessions. This result suggests that sensors (both neurological and peripheral) may be removed and replaced over the course of many interactions with a pBCI system without affecting its performance. Future work on multi-session and multi-day pBCI system use should seek to replicate this (lack of) effect between sessions in other tasks, temporal time courses, and data analytic approaches while also focusing on non-stationarity and variable classification performance due to intrinsic factors.

## Introduction

Practical applications of brain-computer interface (BCI) systems, whether used for direct control or passive monitoring (pBCI; Zander et al., 2010), require stable performance over sustained usage. pBCI performance may be unstable for many reasons, such as changes in the physical properties of the sensors used, the location of sensors, variance in other cognitive states of a participant (e.g., fatigue), and drift or non-stationarity in the signals collected. Non-stationarity in physiological signals can severely hamper routine use of pBCI (Christensen et al., 2012), regardless of the true underlying cause for their non-stationarity. This issue has been addressed in previous BCI work via recalibration of the learning algorithm (e.g., Pfurtscheller and Neuper, 2001) or the use of adaptive algorithms that continually update the mapping between signals and output class (e.g., Vidaurre et al., 2006). Nevertheless, an improved understanding of the source and nature of non-stationarity would support continued improvement in the long-term stability of BCI and pBCI systems. In order to properly explore non-stationarity in the context of pBCI system performance, an essential first step is to rule out sensor and data collection system-related variance. Methodological variability due to electrode replacement could arise from a number of factors including, but not limited to, changes in transducer and sensor properties such as electrode impedance (Ferree et al., 2000), degradation from use and wear (Geddes et al., 1969), technician technique (self or third party), and uncontrolled ambient and environmental conditions. While these issues are unique in their own right, a practical approach is to collapse across all possible factors and consider the act of removing and replacing the electrode array to be encompassing of these nuances and others that may have not been detailed here, as well.

In BCI applications, sustained usage will be most dependent on the ability to demonstrate longitudinal usability over satisfactory periods of time. The time course of declines in pBCI performance suggests that non-stationarity in physiological signals is significant after, at most, a few hours. pBCI system accuracy has previously been observed to decline significantly when training and test data were separated by minutes or hours, but the additional decline when separated by days rather than hours was relatively negligible (Christensen et al., 2012). This time course also suggests that inter-day effects such as consolidation or sleep quality are not likely to be comparatively significant contributors to signal non-stationarity. While this may be a floor effect, the fact that accuracy was still significantly above chance may serve as evidence that cognitive and affective states of interest can be sufficiently mapped by using feature spaces that are observed and aggregated in the learning set over long temporal periods (cross-session learning).

Separating between-session effects with a cognitive or physiological origin from those with a methodological source remains a practically difficult challenge for multi-day studies. However, given the similar performance in pBCI system accuracy between time courses of hours and days, methodological sources of variability may be instantiated as a factor in dual- (or multi-) session, intra-day experimental designs in order to observe any subsequent effects. As in Christensen et al. (2012), a decrease in cross-session BCI system performance as compared to within-session has been observed in other studies with explicit design considerations for multi-session use, most notably in the area of a reactive BCI framework known as rapid visual serial presentation (RSVP; Bigdely-Shamlo et al., 2008; Meng et al., 2012). Thus, the intra-day, dual-session task design is appropriate to investigate methodological variability given similar decreases in system performance at the multi-day time scale. While not the focus of this work, it is noteworthy that cross-session learning paradigms have been successful in mitigating cross-session performance decrements in the RSVP paradigm (Huang et al., 2011) as well as the pBCI paradigm (Christensen et al., 2012).

Electrophysiological methods, both neural and peripheral in origin, have some drawbacks for BCI applications. Electrodes placed on the skin may move relative to the underlying sources; if removed and replaced, the placement may not be identical, resulting in the spatial sampling of a slightly different distribution of electrical potentials. Systems that use gel to provide a conductive, coupling medium at the electrode interface are generally less susceptible to motion-related problems as compared to dry systems since the gel interface allows some electro-mechanical stability (Estepp et al., 2009, 2010). Electrophysiological signals are also dependent on impedance, and impedance at each electrode will inevitably drift due to changes in the skin interface, sweat, and drying of the gel or other electrolyte used. Dry electrode systems are not without similar problems as well, such as physical shifting of the electrode resulting in a decoupling of the hybrid electrical interface with the skin (Estepp et al., 2010) and stabilization of the electrochemical balance between the electrode and skin over time (Geddes and Valentinuzzi, 1973).

Regarding specific electrophysiological methods, electroencephalography (EEG) has been used in many BCI applications for a variety of theoretical and practical reasons (Donchin et al., 2000; Cheng et al., 2002; Wolpaw and McFarland, 2004). EEG is also a relatively practical technology, as it can be portable, inexpensive, noninvasive, and user-acceptable, particularly with systems requiring little or no skin preparation (e.g., Estepp et al., 2009; Grozea et al., 2011; Chi et al., 2012). EEG is also commonly used in pBCI applications for assessing cognitive (Wilson and Fisher, 1995; Gevins et al., 1997; Jung et al., 1997; Lin et al., 2005) and affective (Harmon-Jones and Allen, 1998; Davidson, 2004; Lin et al., 2010) states. Peripheral physiological measures, such as heart period and blink rate (e.g., Veltman and Gaillard, 1998; Wilson and Russell, 2003b) have also been used as sensitive indicators of cognitive workload. Combining both neural and peripheral physiological sources as features in a pBCI context may lead to overall improved system performance when compared to using neural features alone (e.g., Chanel et al., 2009; Christensen et al., 2012); however, the use of fused physiological sources in the context of pBCI systems is relatively underserved compared to those using neural sources only. An emerging trend in BCI research called hybrid BCI (Millán et al., 2010; Pfurtscheller et al., 2010) may be well-suited to exploring beneficial roles for passive cognitive and affective state assessment that incorporates peripheral physiological sources in combination with active and reactive schemas.

While the effects of physiological non-stationarity can be investigated at the individual signal or feature level, another reasonable approach is to study the system behavior at the learning algorithm decision level, or pattern classifier output stage, as it relates to the design of the protocol. A common practice in pBCI system analysis of this type is to reduce the likelihood of results that are unique to any single learning method (e.g., Garrett et al., 2003; Christensen et al., 2012) by investigating a number of varying approaches for the underlying paradigm being studied; this, of course, necessitates an open-loop system design whereby the learning algorithm segment of the system can be substituted *post-hoc* using data collected a priori. While many variants of common learning methods exist in both the BCI and pBCI literature, popular choices include Linear Discriminant Analysis (LDA; for use in cognitive task classification, see Wilson and Fisher, 1995; Berka et al., 2004; Thatcher et al., 2005; for use in traditional active BCI, see Pfurtscheller et al., 1998; Guger et al., 2001; Blankertz et al., 2002; Parra et al., 2005), Support Vector Machines (SVM; e.g., Kaper et al., 2004; Lal et al., 2004; Schlögl et al., 2005; Thulasidas et al., 2006; Sitaram et al., 2007), and Artificial Neural Networks (ANN; for use in cognitive task classification, see Wilson and Russell, 2003a,b, 2007; Christensen and Estepp, 2013; for use in traditional, active BCI applications, see Pfurtscheller et al., 1996; Piccione et al., 2006).

The present work sought to examine the contribution of neural and peripheral physiological sensor (electrode) removal and replacement between sessions in a dual-session task paradigm to learning algorithm performance (the decision-level of the pBCI system) decrement over time. Based on previous work in open-loop (Wilson and Russell, 2003a,b; Estepp et al., 2010; Christensen et al., 2012) and closed-loop (Wilson and Russell, 2007; Christensen and Estepp, 2013) systems analysis, cognitive workload monitoring in a complex, multitask environment was chosen as the state paradigm. Following thorough task training, one set of participants completed two pBCI sessions in a single day without change to their electrode montages while an independent set of participants had their electrode montages removed and replaced with a new set of electrodes between the first and second session. Electrocardiography (ECG) and electrooculography (EOG) data were collected simultaneously with the EEG. Additional subjective state (workload) assessment and task performance data were also collected. Electrode impedances were measured before and after each session. Using a common feature set, k-folded learning trials were performed using four unique learning approaches, thus mitigating the likelihood of spurious results due to any single, *ad-hoc* method or test result. This design enabled direct comparison of between-session classifier accuracy with and without montage replacement, thus quantifying the impact of between-session methodological variability on pBCI performance.

## Materials and methods

### Participants

Twenty participants (13 male, age range of 18–28 years, mean age of 21.45 years) were recruited to participate in this study. This protocol was reviewed and approved by the Air Force Research Laboratory Institutional Review Board and performed in accordance with all relevant institutional and national guidelines and regulations. All prospective participants received a study briefing and completed comprehensive written informed consent prior to their voluntary participation in this study. Participants were compensated for their time unless otherwise employed by the Department of Defense at the time of their participation.

### Between-session electrode preparation: the between-subjects factor

To investigate the effect of methodological variability due to electrode replacement, a between-subjects group factor was introduced between two sessions (S1 and S2) in a dual-session study design. Half of the available participants (10 of 20) were randomly selected to keep their electrode montage in place (referred to as the “Remained” group), while the other half had all electrodes removed and replaced between sessions (referred to as the “Replaced” group). The Replaced group washed and dried their hair (all using the same baby shampoo without conditioner) after having the first set of electrodes removed. Any markings that may have been used to ensure appropriate electrode cap placement prior to S1 were also removed. Prior to the beginning of S2, the electrode montage was reapplied for the “Replaced” group using a different set of electrodes than was used in S1. This procedure was designed to introduce methodological variability due to electrode replacement, if existing, on a shorter time scale than between days such that its potential effects could be reasonably isolated from previously observed between-day effects in learning algorithm performance (Christensen et al., 2012).

### AF-MATB simulation environment

**Figure 1:**
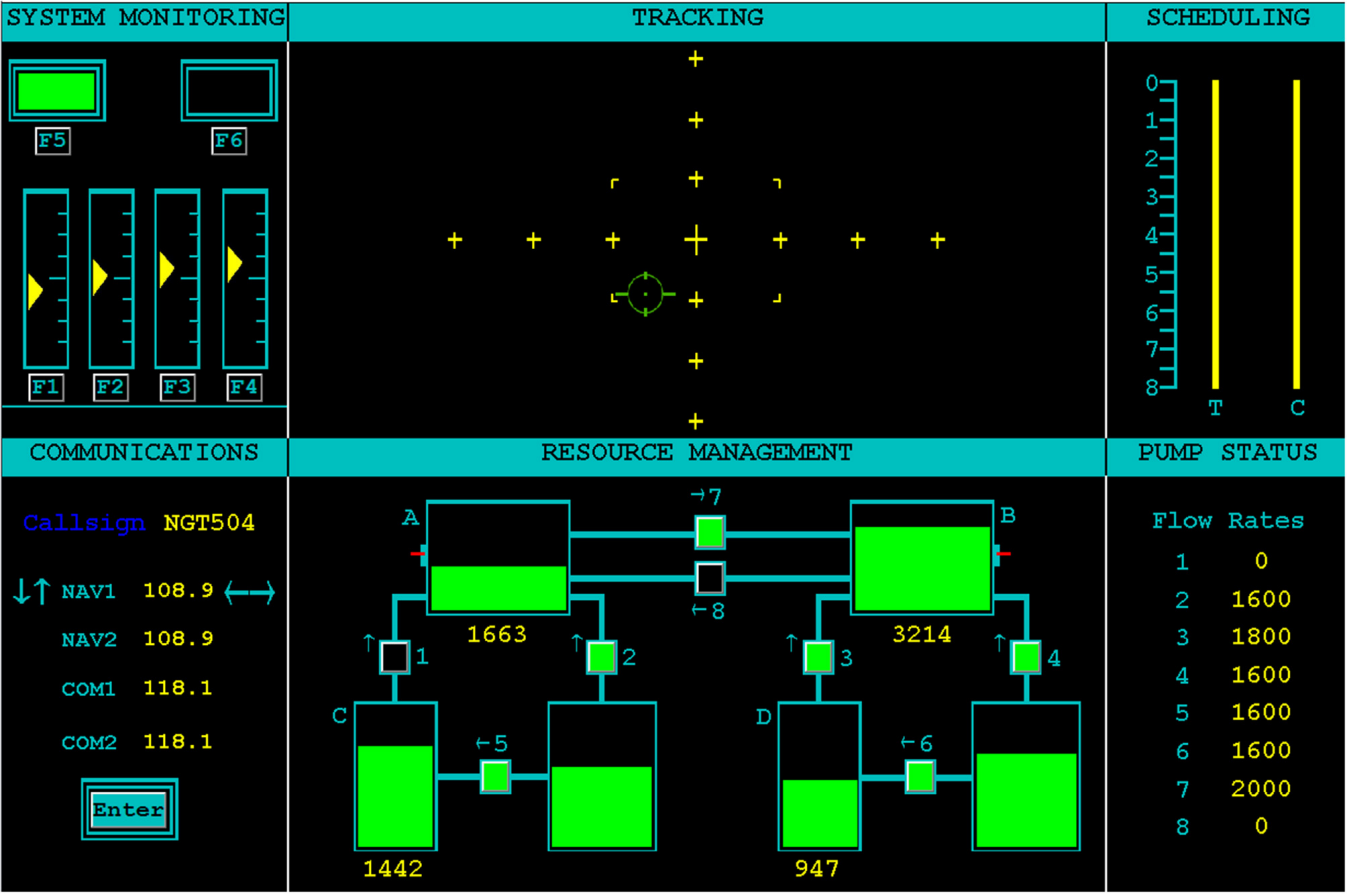
**User interface for the AF-MATB task environment**. The four subtasks (System Monitoring, Tracking, Communications, and Resource Management) are shown in the left and center columns on the interface. The right column shows Scheduling and Pump Status information windows. The Scheduling information window was disabled for this study. More information on the AF-MATB task can be found in AF-MATB User's Guide (Miller, 2010) and in the Supplementary Material for this manuscript.

The Air Force Multi-Attribute Task Battery (AF-MATB; Miller, 2010) was used as a realistic, ecologically-valid multitask environment in which a participant's workload could be varied. The AF-MATB task interface is shown in Figure 1. The task is broadly representative of aircraft operation (particularly remote piloting), and can include compensatory manual tracking, visual and auditory monitoring, and a dynamic resource allocation task. Both AF-MATB and its original instantiation, MATB (Comstock and Arnegard, 1992), have been utilized in numerous studies concerning the use of pBCI architectures and the assessment of cognitive workload in individuals (e.g., Wilson and Russell, 2003b; Christensen et al., 2012) and, when coupled with adaptive automation rule sets, in closed-loop studies (e.g., Freeman et al., 1999; Prinzel et al., 2000, 2003; Wilson and Russell, 2003b). For this study, the visual (System Monitoring) and auditory (Communications) monitoring, compensatory manual tracking (Tracking), and Resource Management tasks were presented simultaneously during all task conditions. The remaining two panels (Scheduling and Pump Status) are informational panels only. Scheduling, although disabled for this study, can be used to convey information about future task state of the Tracking (T) and Communications (C) subtasks. Pump Status displays the current flow rate of the pumps in the Resource Management subtask. For additional details about the AF-MATB simulation environment and its properties, please refer to the online Supplementary Materials for this manuscript.

The demands of each task were varied so that, overall, two levels of individualized difficulty were presented. Participants were trained for a minimum of 2 h per day over 5 different days on AF-MATB until their performance parameters attained asymptote with minimal errors. This procedure helped to reduce learning effects and allowed participants to reach a desired level of familiarity and comfort with the laboratory setting. Task difficulty was increased over the training sessions in order to find a high difficulty level for each individual that met minimum task performance criteria. Participants were not instructed to prioritize any one task over the others. For additional details of the task and training procedure, please see the online Supplementary Materials for this manuscript.

### AF-MATB testing and data collection

On the testing day, participants completed four AF-MATB trials. These trials, each 15 min in length, were divided between two sessions S1 and S2. Trial type within each session was balanced to one each of low and high task difficulty. The order of trials in each session was randomized for all participants. The end of S1 and start of S2 were chronologically separated by 45 min.

**Figure 2:**
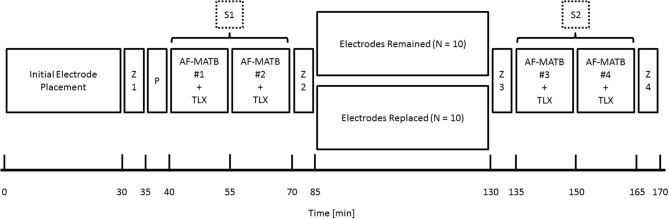
**Experimental timeline for data collection**. Data collection began with initial electrode preparation and placement and an initial measurement of impedance (Z1). A 5-min practice trial (P) was given to participants to re-familiarize themselves with the task interface before beginning data collection. Session 1 (S1) consisted of two, 15-min AF-MATB trials (one at each task difficulty level and paired with a NASA-TLX assessment administered at the end of the trial) followed by a second impedance measurement (Z2). The between-subjects factor of electrode replacement was introduced between the first session and the second session (S2). S2 also consisted of two, 15-min AF-MATB trials bookended by two additional impedance measurements (Z3 and Z4). Order of the AF-MATB trials (with respect to task difficulty) was randomized independently within each session.

Figure 2 depicts an approximate timeline for the data collection. Data collection began with initial electrode preparation and placement and an initial measurement of impedance (Z1). A 5-min practice trial (P) was given to participants to re-familiarize themselves with the task interface before beginning data collection. Session 1 (S1) consisted of two, 15-min AF-MATB trials (one at each task difficulty level and paired with a NASA-TLX assessment administered at the end of the trial) followed by a second impedance measurement (Z2). The between-subjects factor of electrode replacement was introduced between the two sessions. S2 also consisted of two, 15-min AF-MATB trials bookended by two additional impedance measurements (Z3 and Z4).

### Electrophysiological recording

**Figure 3:**
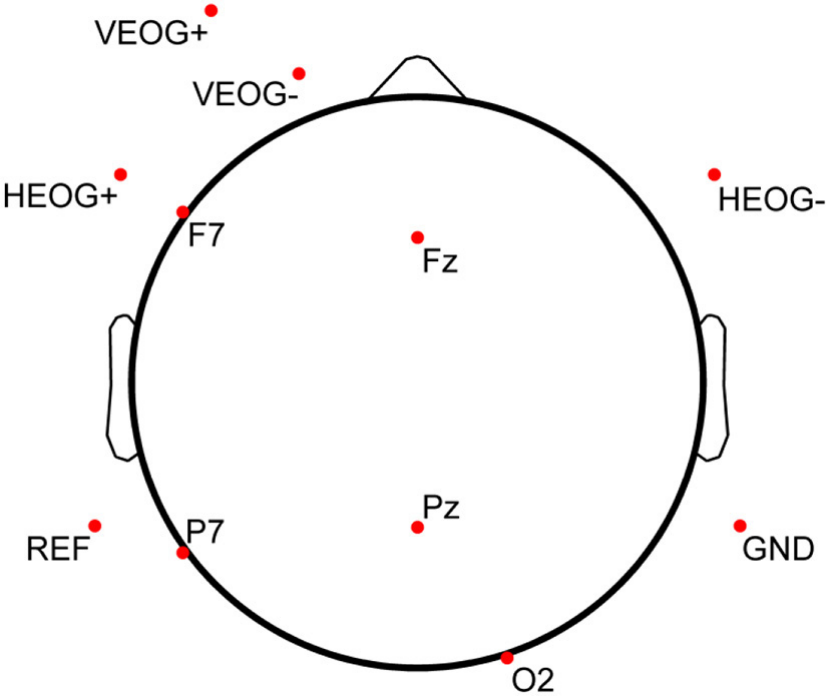
**Electrode montage used for electrophysiological data collection**. Each of the 11 electrodes shown here was a single-lead, 9 mm tin cup electrode. Bipolar lead configuration for ECG [(+) on left clavicle, (−) on sternum] is not shown in this diagram. Electrodes below the horizon of the axial view are shown with a flattened projection perspective.

Prior to completing the practice trial on testing day (the sixth and final day of the protocol), participants were outfitted with a standard elastic fabric EEG electrode cap (Electro-Cap International, Inc., Eaton, OH, USA) containing 9 mm, tin cup electrodes positioned according to the International 10–20 System (Jasper, 1958) and its 10-10 (Chatrian et al., 1985) and 10-5 (Oostenveld and Praamstra, 2001) extensions. The EEG cap was sized and fitted according to measured head circumference above the nasion. After measuring the nasion-to-inion distance, frontal poles (Fp1 and Fp2) were placed at the first 10% distance marker above the nasion, and alignment of Fz was verified to be consistent with the 50% distance markers (nasion-inion and intra-pre-auricular). Five EEG channels on the electrode cap (Fz, F7, Pz, P7, and O2) were used during data acquisition. Matching, single-lead tin cup electrodes were also placed on the outer canthus of each eye (forming a bipolar channel for horizontal EOG, or HEOG), inferior to and superior to the left eye on the orbital bone (forming a bipolar channel for vertical EOG, or VEOG), and on the left (common reference) and right (amplifier ground) mastoid processes. Disposable Ag/AgCl pediatric/neonatal electrodes (Huggables, CONMED Corp., Utica, NY, USA) were positioned on the left clavicle and sternum, forming a bipolar channel for ECG. All peripheral channels were prepared by cleaning the skin with 70% isopropyl alcohol prep pads and gently scrubbing the cleaned surface with NuPrep (Weaver and Company, Aurora, CO, USA). EEG scalp sites were prepared via syringe with a blunted needle and then filled with Electro-Gel (Electro-Cap International, Inc., Eaton, OH, USA). The full electrode montage is displayed in Figure 3 (electrodes below the horizon of the axial view are shown with a flattened projection perspective). All electrophysiological channels were chosen based on a previous saliency analysis and sensor downselect from a similar study using the MATB task environment (Russell and Gustafson, 2001).

A BioRadio 110 (Great Lakes NeuroTechnologies, Cleveland, OH, USA) telemetry system was used to acquire the 8 aforementioned channels of electrophysiological data (using a common reference montage for the five EEG channels) during task performance. All available channels were recorded at 200 Hz, with 12-bit resolution, using an AC-coupled amplifier (bandpass filtered between 0.5 and 52.4 Hz).

### Subjective workload assessment

Participants' subjective workload ratings were assessed using the National Aeronautics and Space Administration's Task Load Index (NASA-TLX; Hart and Staveland, 1988). The NASA-TLX was administered immediately following each of the four AF-MATB trials (Figure 2). Participants completed both the individual six subscale ratings as well as the Sources of Workload subscale comparison.

### Electrode impedance

Complex electrode impedance was monitored and recorded pre- and post-session for both S1 and S2. This was done to quantify changes in impedance during and between sessions, regardless of whether the recording system was replaced or left in place. Upper limit thresholds for accepting an electrode preparation were 5 kΩ for EEG and 20 kΩ for EOG and ECG electrodes. Electrodes were re-prepped during the pre-session impedance check if any of these thresholds were exceeded.

### Electrophysiological data processing

**Figure 4:**
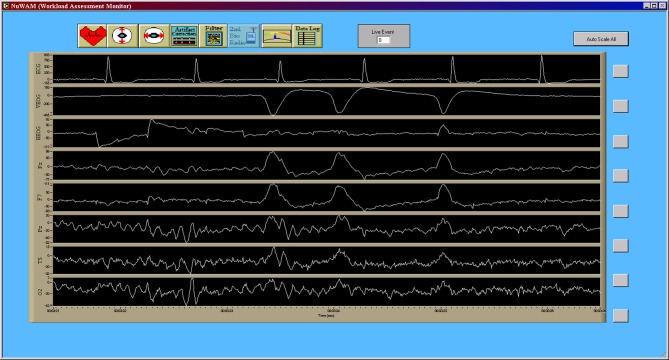
**Primary data collection user interface for the custom data acquisition software**. This interface allows researchers to view the raw time series, in real-time, during data acquisition in order to monitor data quality. Note that the seventh channel, T5, is the original convention presented by Jasper (1958) for what is now commonly referred to as P7 (Chatrian et al., 1985; Oostenveld and Praamstra, 2001). Note that all data from the data acquisition software (Figures 4–**7**) are shown using a “playback” feature of the software that allows for simulated real-time, *post-hoc* viewing and reprocessing of the original raw data time series using the feature processing pipeline. Y-axis units of all time series are given in microvolts [uV].

All preprocessing and generation of electrophysiological feature data was accomplished in real-time as part of the data acquisition in a software suite developed in the LabVIEW (National Instruments Corporation, Austin, TX, USA) development environment (Krizo et al., 2005). The primary user interface for this software is shown in Figure 4. All feature data, as well as the raw electrophysiological data, were saved for further *post-hoc* (offline) processing. Electrophysiological feature data were created using an averaging window with an overlap to define the rate at which this data was updated. This update rate was synchronized to 1 Hz across all feature types. A total of 37 features consisting of EEG, VEOG, and HEOG band powers, inter-beat interval (IBI) between consecutive R-wave peaks of the ECG, and blink rate derived from the VEOG channel were used in this study.

#### EEG data processing

**Figure 5:**
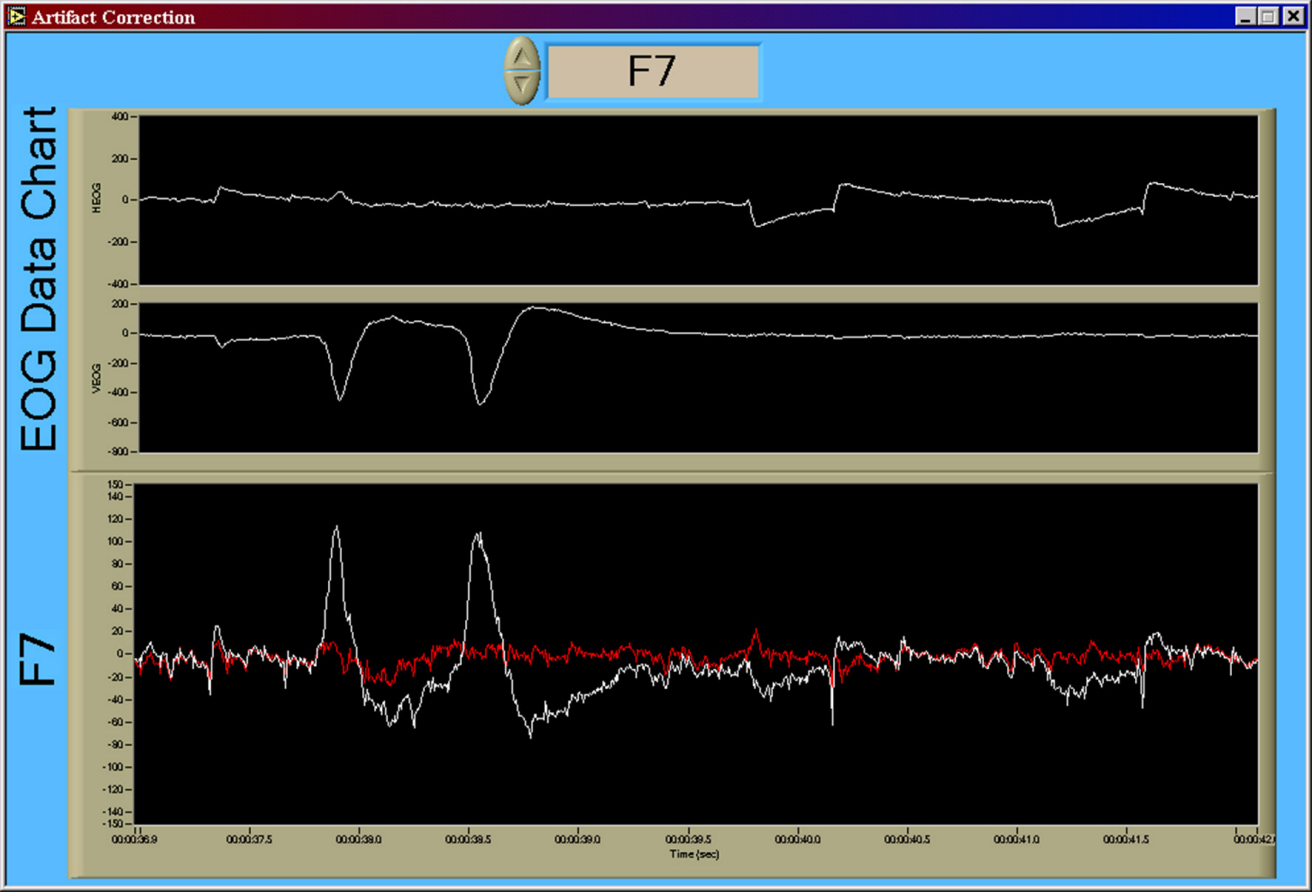
**Result of the recursive least-squares noise canceling adaptive filter**. Using the implementation of He et al. (2004, 2007), VEOG and HEOG bipolar time series are used as reference noise input channels to the adaptive noise canceling structure. The time series shown in this figure, in order from top to bottom, are HEOG, VEOG, and F7 (in white). The output of the adaptive filter is shown in red. Large amplitude artifact from blink activity (early in the time series) and saccadic activity (later in the time series) are absent in the noise-corrected time series. Y-axis units of all time series are given in [uV].

EEG channels were first corrected for gross artifact due to eye movement using a recursive least-squares implementation of a noise canceling adaptive filter (He et al., 2004, 2007). An example of the original and noise-canceled time series for F7 is shown in Figure 5. Following ocular artifact correction each EEG channel was then used to create power spectral densities (PSD) via the Discrete Fourier Transform (DFT) algorithm with a corresponding Hanning window (also known as the periodogram method) over a 1 s window. Band power estimates were then derived from the PSD of each of the channels using commonly defined traditional clinical frequency bands. The frequency band ranges used in this pipeline were: delta (0.5–3 Hz), theta (4–7 Hz), alpha (8–12 Hz), beta (13–30 Hz), and gamma (31–42 Hz). EEG features were created by averaging these 1 s band power estimates over a 10 s window (with a 9 s overlap) and then applying a base 10 logarithmic transform to improve the normality of the band power feature distributions (Gasser et al., 1982). This resulted in 25 features (5 EEG channels × 5 frequency bands) from the EEG data.

#### EOG data processing

**Figure 6:**
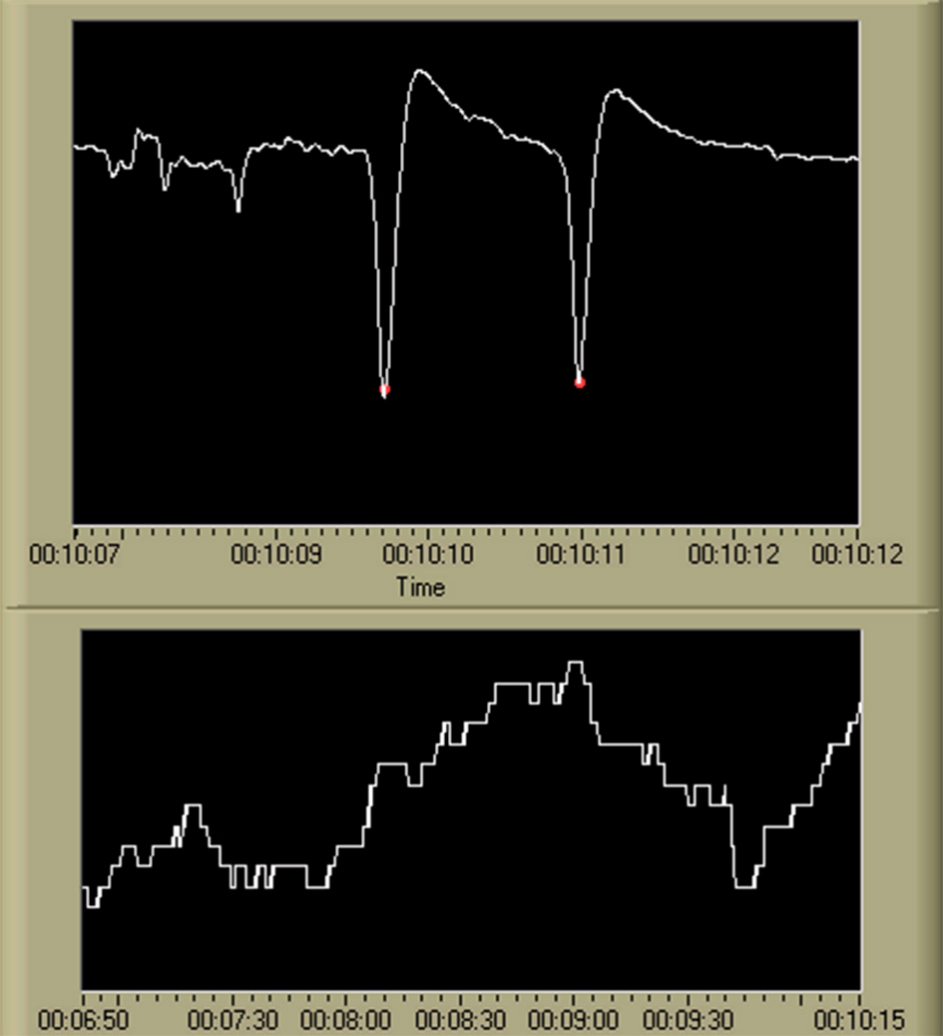
**Output of the blink detection algorithm (Kong and Wilson, 1998) and the resulting blink rate time series**. Detected blinks are shown in the first chart using red marking indicators at the apex of each blink. The second chart displays the resulting blink rate time series. While not shown on the display, the scale for the VEOG time series is [uV], and the scale for the blink rate time series is [blinks/min]. The time scale is in standard HH:MM:SS format.

VEOG and HEOG channels were processed using the same frequency band pipeline as the EEG data. This resulted in an additional 10 frequency band features (2 EOG channels × 5 frequency bands). VEOG was also used in a real-time implementation of a blink detection algorithm (Kong and Wilson, 1998). Blink counts were summed over 30 s window (with a 29 s overlap) to calculate average blink rate as a feature (blinks per [min]). In total, 11 additional features were derived from the VEOG and HEOG channels. An example of the output of this algorithm, as well as the resulting blink rate feature time series, is shown in Figure 6.

#### ECG data processing

**Figure 7:**
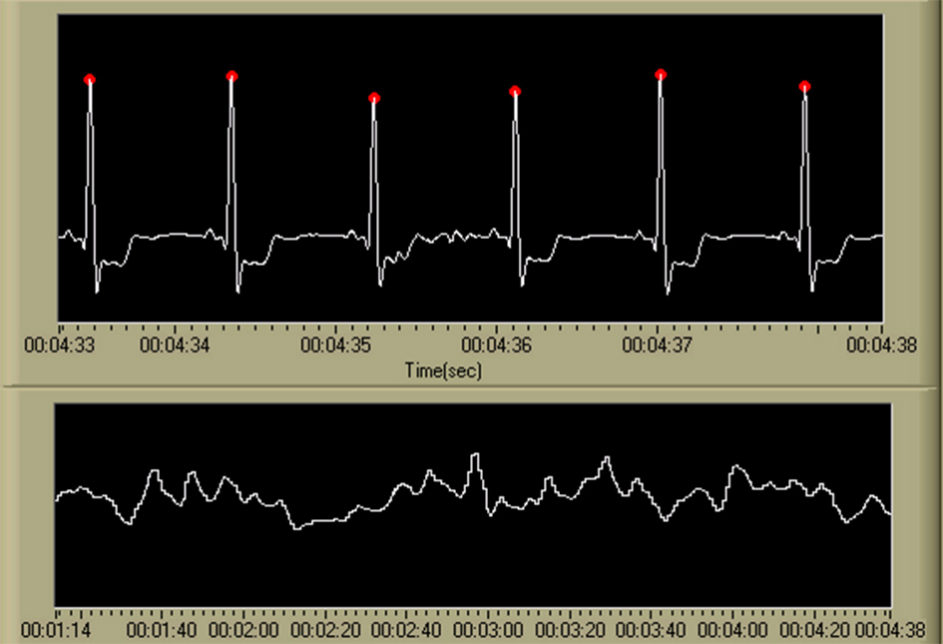
**Output of the R-wave detection algorithm (Pan and Tompkins, 1985; Hamilton and Tompkins, 1986) and the resulting IBI time series**. Detected *R*-waves are shown in the first chart using red marking indicators at the apex of each QRS complex. The second chart displays the resulting IBI time series. While not shown on the display, the scale for the ECG time series is [uV], and the IBI time series is visualized as heart rate in beats per minute, or [BPM]. The time scale is in standard HH:MM:SS format.

A single feature, related to heart rate, was derived from the ECG data. Individual cardiac cycles, as defined by the R-wave, were first identified using a real-time algorithm developed by Pan and Tompkins (1985) and Hamilton and Tompkins (1986). The IBI time series within a 10 s window (with a 9 s overlap) was averaged to create the IBI feature. An example of the output of this algorithm, as well as the resulting IBI feature time series, is shown in Figure 7.

### Machine learning approaches

The pBCI framework in this study consisted of using data from the first session on the test day (S1) to train a machine learning algorithm that could then be used as a fixed pattern classifier to assess the participants' cognitive workload in the second session (S2). This general architecture supports workload assessment in real-time by providing the feature vectors, updated at 1 Hz, as the input layer of the learning algorithm. A number of learning approaches were compared in investigating whether the between-subjects factor of electrode removal and replacement affects learning algorithm accuracy in the simulated real-time assessment of workload during S2. Each learning algorithm was structured to solve a binary classification problem of low vs. high workload (a priori hypothesized to be driven by low vs. high task difficulty, but which can be tested *post-hoc* with a combination of task performance and subjective workload assessment measures). All learning approaches were implemented in *post-hoc* analyses using the feature vectors that were generated by the real-time acquisition and processing software (although the feed-forward implementation of each learning algorithm could be integrated into the real-time software). All *post-hoc* implementations of the learning approaches used in this study were developed in MATLAB R2010b (The Mathworks, Inc., Natick, MA, USA) using custom-written code and available toolboxes where noted.

#### Accuracy vs. sensitivity

While the overall accuracy of the learning approach (represented as proportion of 1 s epochs correctly classified as either low or high workload) is a useful measure to help understand algorithm performance, other measures such as d' (d-prime; Green and Swets, 1966) may be better suited for quantified performance comparisons. The use of d' as a signal detection sensitivity measure may be preferred as it is free of bias that may occur in using the proportion of epochs correct as an algorithm performance measure (such as the case would be if algorithm performance was biased toward one class in the binary problem). The calculation of d' is given in Equation (1), where z() represents the inverse of a unit normal Gaussian cumulative distribution function (the “norminv” function in MATLAB, with μ = 0 and σ^2^ = 1), and True Positive Rate (TPR) and False Positive Rate (FPR) are calculated from the test set confusion matrix and have a range of (0,1) (not inclusive).

(1) $$d^{\prime }=z\left(TPR\right)-z\left(FPR\right)$$

In this work, the correct detection of a high workload state epoch is considered to be a true positive (TP), while any low workload state epoch incorrectly classified as being from a high workload state is a false positive (FP). TPR and FPR are then calculated from Equations 2 and 3 using the confusion matrix structure that is shown in Table 1.

(2) $$TPR=\{\begin{array}{cc} \frac{1}{\left(TP+FN\right)}, & \;TP=0\\ \frac{TP-1}{TP+FN}, & \;FN=0\\ \frac{TP}{TP+FN}, & \;otherwise \end{array}$$

(3) $$FPR=\{\begin{array}{cc} \frac{1}{\left(FP+TN\right)}, & \;FP=0\\ \frac{FP-1}{FP+TN}, & \;FN=0\\ \frac{FP}{FP+TN}, & \;otherwise \end{array}$$

**Table 1:** **Classifier output structure (confusion matrix) used to determine d′**.

		**Estimated class**	
		**High workload**	**Low workload**
**Truth class**	**High workload**	True positive (TP)	False negative (FN)
	**Low workload**	False positive (FP)	True negative (TN)

#### Definition of learning set, test set, and k-fold procedures

All learning algorithms were trained using data from S1 (the learning set) and then feed-forward tested on S2 (the between-session test set) to create an unbiased estimate of learning algorithm performance. To guard against spurious learning results, a k-fold (*k* = 10) cross-validation procedure was used on the learning set. Each set (learning and test) contained approximately 1800 balanced samples (900 from each of the 15-min low and high task difficulty trials in the session) as a product of the 1 Hz feature vector update rate. The 10 folds were created by randomly subsampling 90% of the available data from S1, resulting in approximately 1620 learning samples in each fold. Approximately 162 samples, or 10%, of the learning set was reserved as a nested test set to observe the within-session performance of the learning approach. Learning algorithm output for the within-session and between-session test sets was unweighted (not explicitly biased) given balanced classes in the learning set. This folding process, per the Central Limit Theorem, will result in a normal distribution for classifier performance (expressed as sensitivity, or d') for a sufficient number of folds, thus ensuring equality of the mean and median of each performance distribution. Due to the number of folds used in this analysis (*k* = 10), the median of each set of folds is used to represent that learning approach's performance in all subsequent analyses of variance. The choice of median in this analysis is sufficient to reduce any distribution skew resulting from the *k* = 10 folds that would otherwise bias the distribution mean.

The learning set was normalized to itself by converting (within-feature) to z-scores using the mean and standard deviation of each of the 37 features separately for each participant. These mean and standard deviation vectors were then used to z-score the within-session nested test sets from S1 and the between-session independent test sets from S2 in order to simulate a real-time implementation of the feed-forward algorithm architecture.

#### Linear discriminant analysis

LDA was implemented via the MATLAB Statistics Toolbox v7.4 (R2010b) using an implementation of the “classify” function. LDA defines a linear decision boundary based on linear combinations of the input feature vectors to separate the learning set according to categorical class labels. Classification is then achieved by assigning the estimated class of each tested sample according to its location as referenced to the linear decision boundary.

#### Support vector machines

The implementation of the SVM in this study utilized the kernel approach to mapping the learning set to a non-linear feature space. Lacking any a priori decision information to choose an appropriate kernel for this particular dataset, two popular approaches were tested: a linear kernel (LIN) and a (Gaussian) radial basis function (RBF) kernel. Kernel parameters for both the LIN and RBF kernels were optimized via the “tunelssvm” function using the multidimensional unconstrained non-linear optimization approach (“simplex”) contained within the LS-SVMLab v1.8 Toolbox (De Brabanter et al., 2010). Both the linear kernel SVM (LIN-SVM) and the radial basis function SVM (RBF-SVM) algorithms were implemented via the exact incremental learning and adaptation approach (Cauwenberghs and Poggio, 2001) with the Incremental SVM Learning in MATLAB package (Diehl and Cauwenberghs, 2003). Following the decision boundary rule for the LDA, classification using both the LIN-SVM and RBF-SVM was achieved by assigning the estimated class of each tested sample according to is location as referenced to the non-linear decision boundary.

#### Artificial neural networks

The particular ANN implementation used for this work follows that in Christensen et al. (2012). The input layer of the ANN was matched to the 37 features; a single hidden layer utilized a fully-connected structure. Training was accomplished via the backpropagation algorithm (Lippmann, 1987; Widrow and Lehr, 1990). A nested validation set (33% of the learning set) was used to implement an early stopping rule (del R Millan et al., 2002) at the learning iteration at which root mean-squared error, or RMSE, was minimized for the validation set. This early stopping rule was intended to guard against overfitting to the learning set (Wilson and Russell, 2003a; Bishop, 2006). The ANN used a 2-node output layer for the binary classification problem addressed here. Binary classification was implemented by assigning each test case to the higher weight between the 2-node outputs. This ANN structure was implemented in MATLAB R2010b using custom-developed code and functions from the Neural Network Toolbox v7.0 (R2010b).

## Results

While the main factor being investigated in this work is the (between-subjects) effect of electrode removal and replacement on learning algorithm accuracy in a pBCI framework for cognitive workload assessment, a number of analyses must first be accomplished given factors of task difficulty (two levels, low and high) and session (two levels, S1 and S2). These two within-subjects factors, when combined with the between-subjects factor of electrode replacement (two levels, Remained and Replaced), were the basis for analyses of both task performance and subjective workload data. In addition, impedance data were analyzed for any significant changes across time (pre- and post-session) and with respect to the between-subjects factor of electrode replacement. Unless noted otherwise all statistical tests were performed using IBM SPSS Statistics Standard 21. All analyses of variance were analyzed using α = 0.01.

### AF-MATB performance

The four primary subtasks in AF-MATB all have associated outcome measures related to task performance. While there exist a number of performance measures for each subtask that could be investigated, a single measure related to “hit rate” appropriate for each subtask was chosen. These measures were: (1) proportion of stimuli (including both lights and gages) with correct responses for the System Monitoring subtask, (2) proportion of stimuli for the participants' active callsign for which the participant responded with a comm channel/frequency change for the Communications subtask, (3) RMS tracking error (from center, in pixels) for the Tracking subtask, and (4) deviation from the nominal fuel level, averaged between Tanks A and B, for the Resource Management subtask. To investigate these multiple task performance measures for this study design, a 2 (task difficulty, within) × 2 (session, within) × 2 (electrode replacement, between) mixed-model multivariate analysis of variance (MANOVA) was performed using the four subtask performance measures as dependent variables.

**Figure 8:**
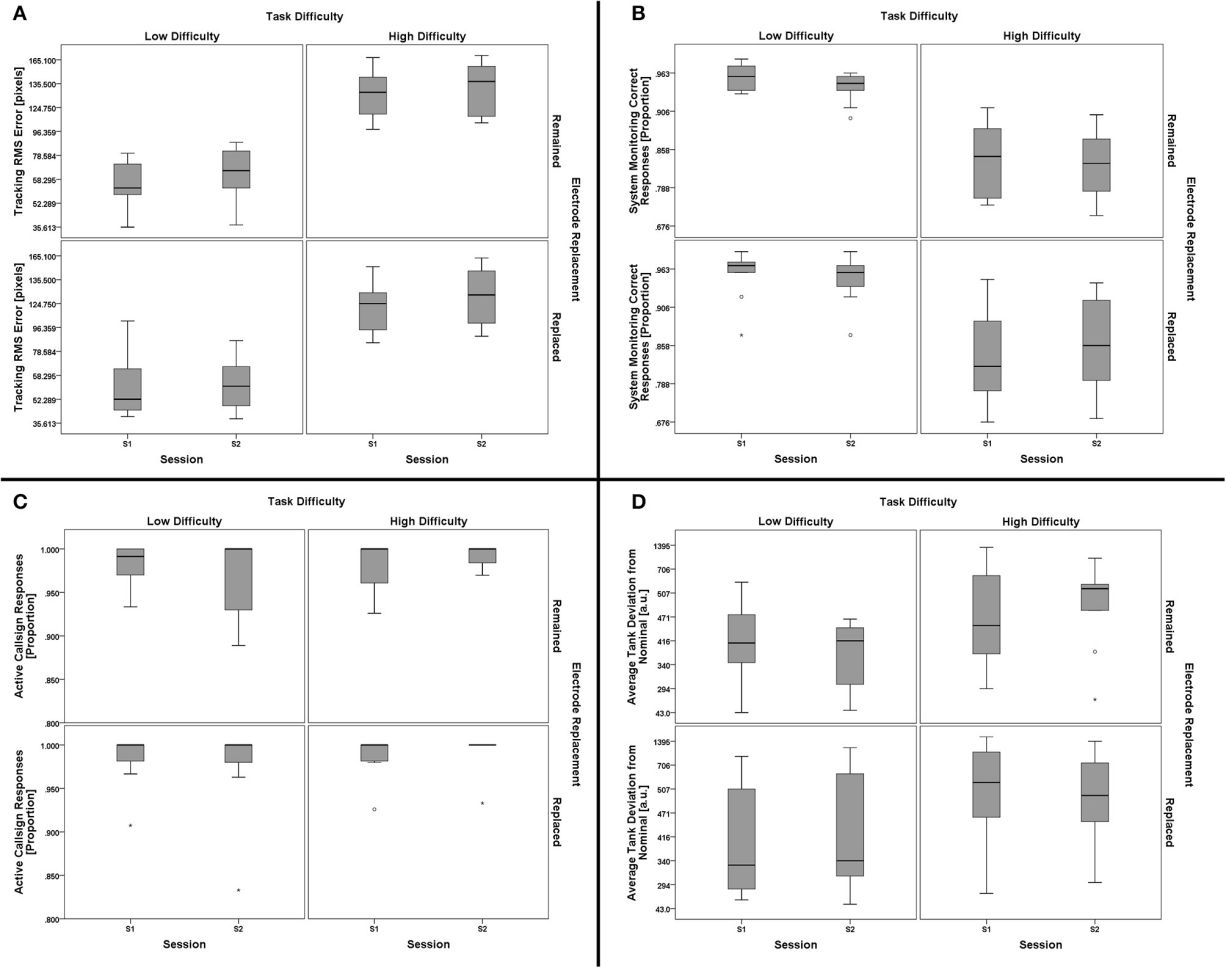
**Boxplots showing AF-MATB subtask performance data representative of the 2 (task difficulty, within) × 2 (session, within) × 2 (electrode replacement, between) mixed model MANOVA results**. Individual subtask performance measures are shown for the Tracking **(A)**, System Monitoring **(B)**, Communications **(C)**, and Resource Management **(D)** subtasks. The only significant effect from all main effect and interaction tests was a main effect of task difficulty on AF-MATB performance. Subtask factors contributing to the main effect of task difficulty on AF-MATB performance were not subjected to further analysis given the goals of this work, but it is clear from the boxplot data that Tracking **(A)** and System Monitoring **(B)** task performances were affected by task difficulty. There appears to be some effect of task difficulty on the Resource Management **(D)** subtask, although individual participant performance in this subtask appears to be more variable than in others. There is no clear difference in Communications **(C)** subtask performance with respect to task difficulty. Otherwise, task performance for both groups (Remained and Replaced) across both sessions (S1 and S2) was consistent, respective of task difficulty. The boxplots shown represent the median (line inside the box), first and third quartiles (bottom and top of the box, or the lower and upper hinges, respectively), and minimum and maximum values (lower and upper whiskers, respectively, or inner fences). Outliers exceeding 1.5 times the box height are shown as individual sample points (circles). Extreme outliers, or those samples exceeding 3 times the box height, are indicated by asterisks.

There was no significant effect of the between-subjects factor of electrode replacement, *F*_(4, 15)_ = 0.890, *p* = 0.494, η^2^_*p*_ = 0.192. For the within-subjects factors, there was no significant main effect of session, *F*_(4, 15)_ = 1.513, *p* = 0.248, η^2^_*p*_ = 0.288, but the main effect for task difficulty was significant, *F*_(4, 15)_ = 104.693, *p* < 0.001, η^2^_*p*_ = 0.965. Two-way interactions for (task difficulty × session), *F*_(4, 15)_ = 2.321, *p* = 0.104, η^2^_*p*_ = 0.382, (task difficulty × electrode replacement), *F*_(4, 15)_ = 1.999, *p* = 0.146, η^2^_*p*_ = 0.348, and (session × electrode replacement), *F*_(4, 15)_ = 1.035, *p* = 0.421, η^2^_*p*_ = 0.216, were all non-significant. The three-way interaction, (task difficulty × session × electrode replacement), was not significant, *F*_(4, 15)_ = 1.030, *p* = 0.424, η^2^_*p*_ = 0.215. Since the exact subtask factors responsible for the main effect of task difficulty are not of importance to the goals of this work, further analysis of subtask effects are omitted in favor of the individual subtask boxplots shown in Figure 8. The significant main effect of task difficulty on performance validates that the task manipulation was successful at inducing significantly different task performance states; the lack of a significant effect of session suggests that performance was consistent across sessions. Similarly, the electrode replacement factor was not of significant effect, thus confirming a lack of difference in task performance between the two groups.

### NASA-TLX

Subjective workload ratings obtained via the NASA-TLX were analyzed in a similar manner to the performance data with very similar results. The factor-weighting procedure per the original work of Hart and Staveland (1988) was used to calculate the overall subjective workload rating for each trial. A 2 (task difficulty, within) × 2 (session, within) × 2 (electrode replacement, between) mixed-model ANOVA was performed to investigate effects of subjective workload.

**Figure 9:**
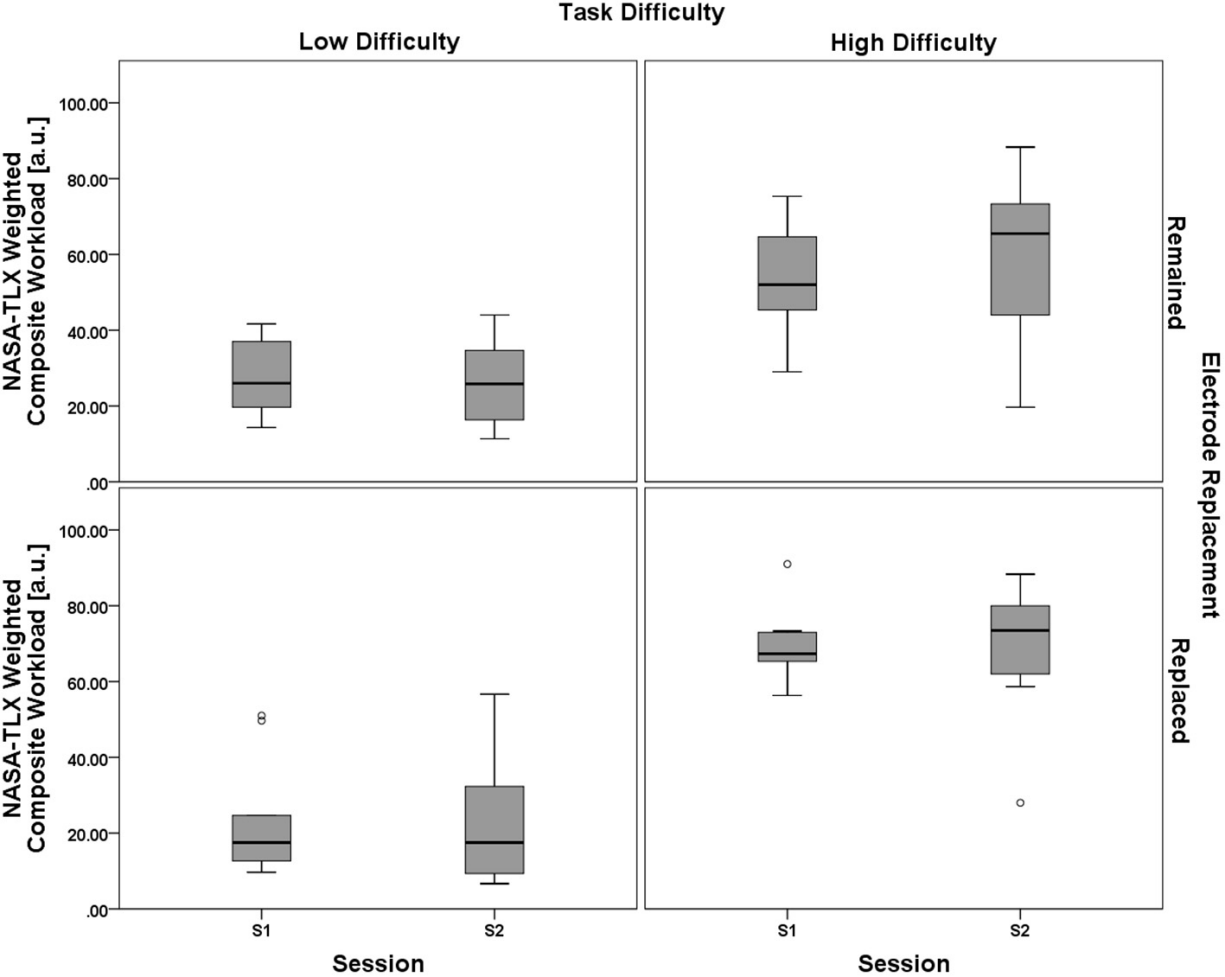
**Boxplots showing NASA-TLX Weighted Composite Workload scores representative of the 2 (task difficulty, within) × 2 (session, within) × 2 (electrode replacement, between) mixed model ANOVA results**. The only significant effect from all main effect and interaction tests was a main effect of task difficulty on NASA-TLX subjective workload ratings. Results of the subjective workload assessment confirm that there was a significant workload difference between the two task difficulty conditions. Otherwise subjective workload for both groups (Remained and Replaced) across both sessions (S1 and S2) was consistent, respective of task difficulty. The boxplots shown represent the median (line inside the box), first and third quartiles (bottom and top of the box, or the lower and upper hinges, respectively), and minimum and maximum values (lower and upper whiskers, respectively, or inner fences). Outliers exceeding 1.5 times the box height are shown as individual sample points (circles). Extreme outliers, or those samples exceeding 3 times the box height, are indicated by asterisks.

Results from the ANOVA test showed no significant main effect of the between-subjects electrode replacement factor, *F*_(1, 18)_ = 0.729, *p* = 0.405, η^2^_*p*_ = 0.039. The within-subject main effect of session was not significant, *F*_(1, 18)_ = 0.269, *p* = 0.611, η^2^_*p*_ = 0.015, but there was a significant main effect of task difficulty, *F*_(1, 18)_ = 66.272, *p* < 0.001, η^2^_*p*_ = 0.786. Two way interactions for (task difficulty × session), *F*_(1, 18)_ = 3.143, *p* = 0.093, η^2^_*p*_ = 0.149, (task difficulty × electrode replacement), *F*_(1, 18)_ = 2.902, *p* = 0.106, η^2^_*p*_ = 0.139, and (session × electrode replacement), *F*_(1, 18)_ = 0.857, *p* = 0.367, η^2^_*p*_ = 0.062, were all non-significant. The three-way interaction, (task difficulty × session × electrode replacement), was also non-significant, *F*_(1, 18)_ = 2.447, *p* = 0.135, η^2^_*p*_ = 0.120. A boxplot showing the NASA-TLX data is shown in Figure 9. As with the analysis of the performance data, the subjective workload data provides additional evidence for the validity of the task difficulty manipulation as a strategy for creating varying workload states that were constant between sessions and groups.

### Electrode impedance (Z)

Four measurements of individual electrode impedance (pre- and post-session for both S1 and S2) were made during this study to account for any change in group-level impedance with respect to the between-subjects factor of electrode replacement. A 4 (measurement time point, within) × 2 (electrode replacement, between) mixed model analysis of variance (ANOVA) was performed to assess any possible impedance changes due to these two factors. Lacking any a priori evidence for investigating each electrode independently, an omnibus measure of impedance was created for each measurement time point by averaging impedance across all electrodes.

**Figure 10:**
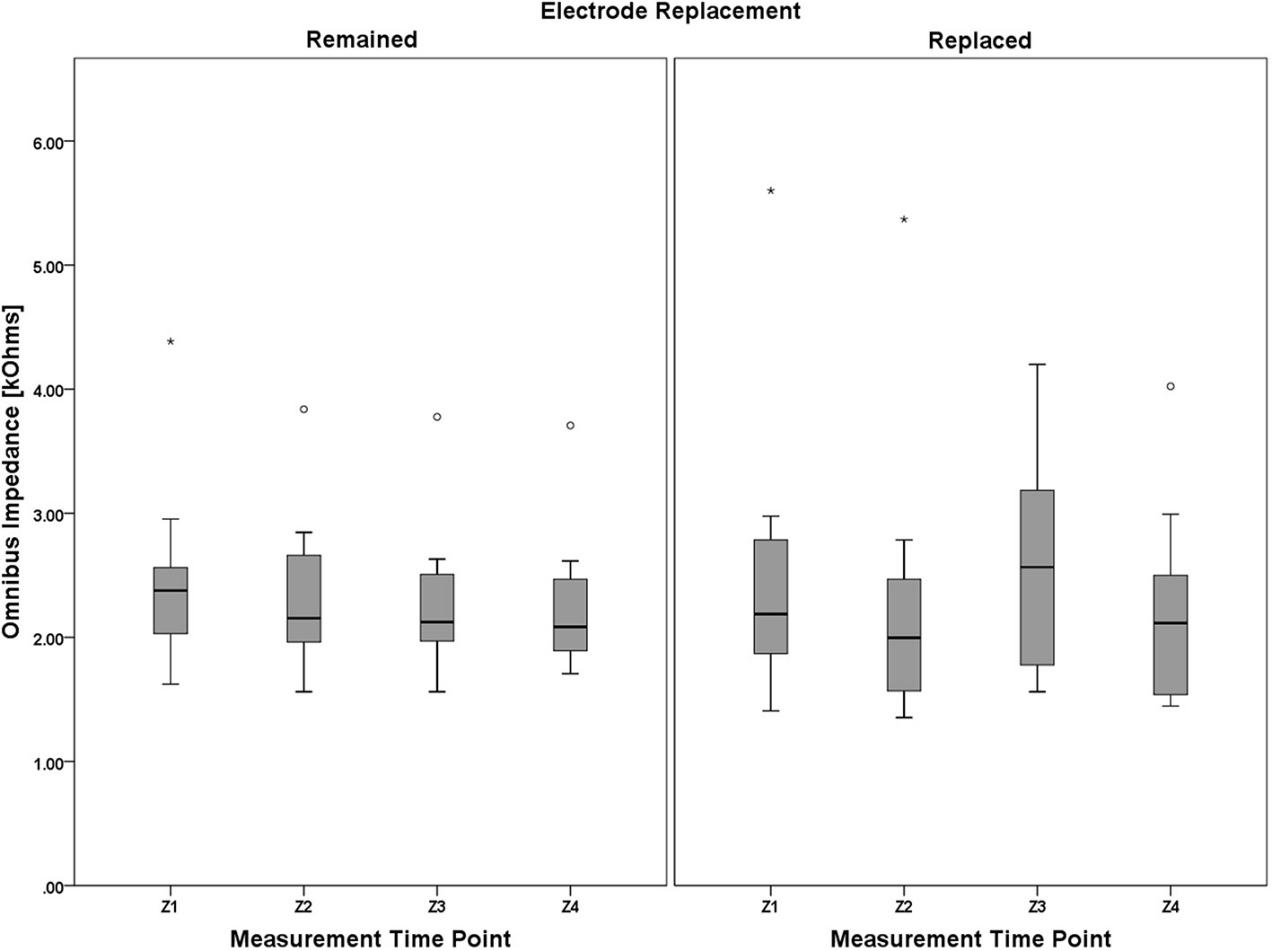
**Boxplots showing omnibus impedance data representative of the 4 (measurement time point, within) × 2 (electrode replacement, between) mixed model ANOVA results**. It is worth noting that, despite the lack of main effect of measurement time point and electrode replacement, or a significant interaction between the two factors, (1) impedance for the Remained group appears to very slightly decrease over the course of the protocol, and (2) this decreasing trend was interrupted by electrode replacement in the Replaced group (although still apparent between Z1/Z2 and Z3/Z4 pre-post session measurement pairs). Electrode impedance was held below an acceptable tolerance for both the Remained and Replaced groups. The boxplots shown represent the median (line inside the box), first and third quartiles (bottom and top of the box, or the lower and upper hinges, respectively), and minimum and maximum values (lower and upper whiskers, respectively, or inner fences). Outliers exceeding 1.5 times the box height are shown as individual sample points (circles). Extreme outliers, or those samples exceeding 3 times the box height, are indicated by asterisks.

Mauchly's test revealed a significant deviation from the assumption of sphericity, χ^2^(5) = 38.397, *p* < 0.001, thus necessitating adjustments to the degrees of freedom. Following the guidance of Huynh and Feldt (1976), the Greenhouse-Geisser estimate of sphericity (Greenhouse and Geisser, 1959) was used ($\hat{\varepsilon }$ = 0.461) in lieu of the Huynh-Feldt estimate ($\tilde{\varepsilon }$ = 0.514) given $\hat{\varepsilon }$ < 0.75. There was not a significant main effect of electrode replacement, *F*_(1, 18)_ = 0.056, *p* = 0.815, η^2^_*p*_ = 0.03, but the main effect of measurement time point approached significance, *F*_(1.382, 24.877)_ = 3.224, *p* = 0.073, η^2^_*p*_ = 0.152. The 2-way interaction, (measurement time point × electrode replacement), was not significant, *F*_(1.382, 24.877)_ = 2.318, *p* = 0.151, η^2^_*p*_ = 0.106. A boxplot depicting the omnibus impedance data is shown in Figure 10. Results of the analysis of the impedance data suggest that impedance for all participants, regardless of electrode replacement group assignment, was constant over the duration of the data collection.

### Example feature data from replaced group

**Figure 11:**
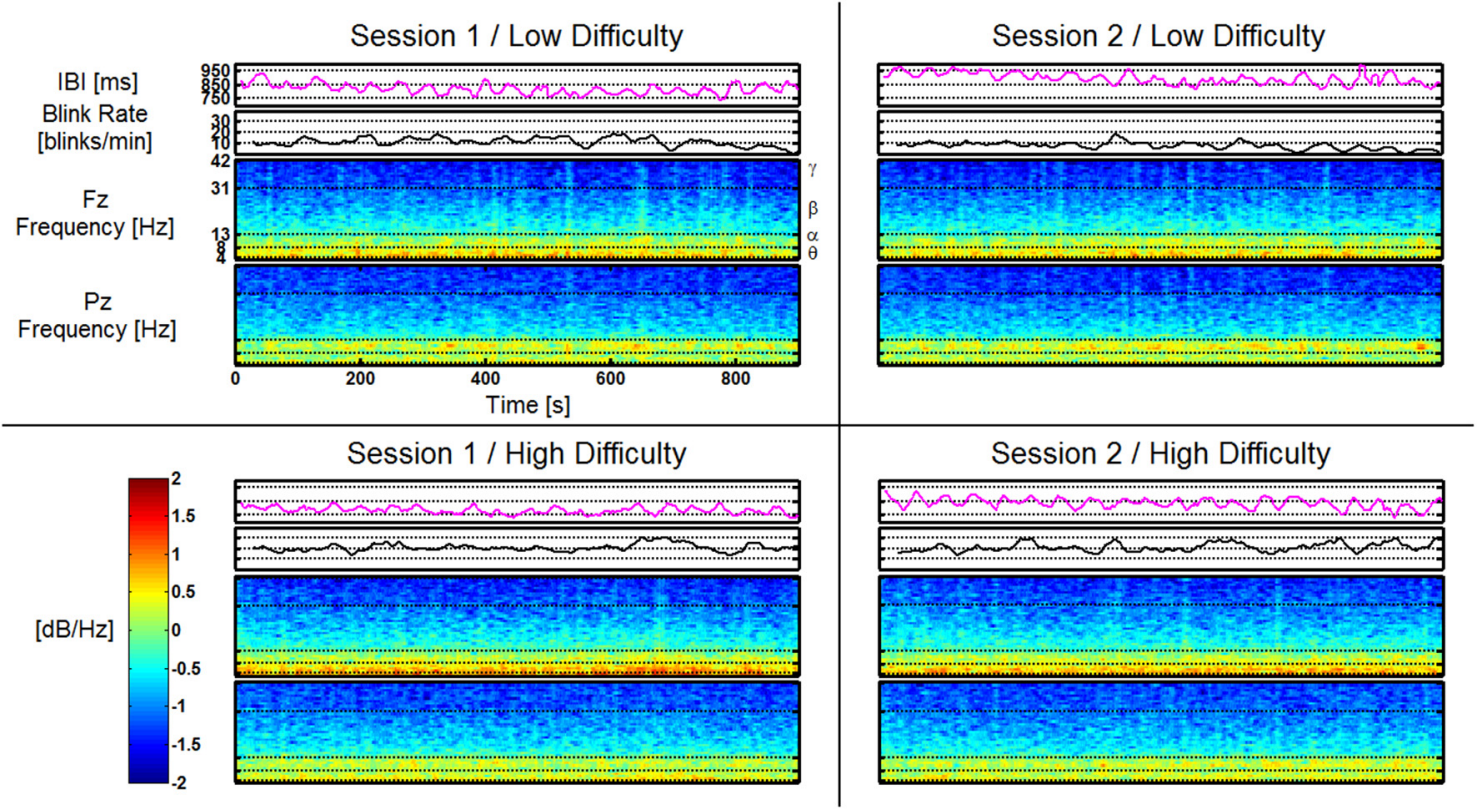
**Feature plots for IBI, Blink Rate, Fz, and Pz (shown as time-frequency plots) for a participant in the Replaced group**. Feature plots for this participant are shown, respective of task difficulty (in rows) and session (in columns). All corresponding feature plots are shown on the same y-axis scale (e.g., all time-frequency plots for Fz and Pz are shown using the scale depicted on the included colorbar). Frequency band ranges are shown on the time-frequency plots. Effects of task difficulty for this participant are clearly visible in Blink Rate, Fz theta, and Pz alpha. There are no visually noticeable effects of session, or as is the case for this participant, electrode replacement.

An example dataset from the Replaced group is shown in Figure 11. The data from this participant is represented as a single time series for both the Blink Rate and IBI features as well as time-frequency plots for both Fz and Pz. All four AF-MATB trials are shown individually in a representation of the 2 (session) × 2 (task difficulty) study design. Like the feature vectors, the data in this figure are averaged using a 10 s window with a 9 s overlap. All corresponding data series are shown on the same scale (e.g., all of the time-frequency plots use the same scale for mapping log power [dB/Hz] to the colormap shown in the colorbar). Individual band ranges for theta, alpha, beta, and gamma are annotated on the time-frequency plots (delta is omitted). Examining the time series, we observe workload differences consistent with results in similar previous studies (Wilson and Fisher, 1995; Gevins et al., 1998), but no obvious differences as a function of session or having the electrodes replaced between sessions.

### Learning algorithm performance

**Figure 12:**
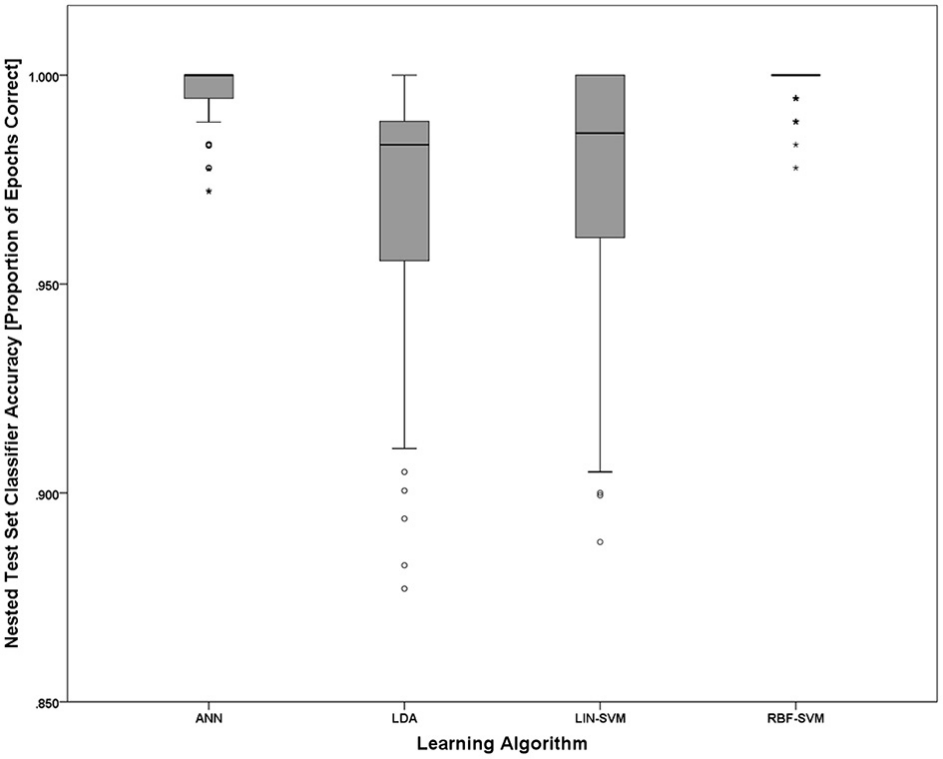
**Nested test set accuracy (withheld from S1) for each of the four learning approaches**. Noting the y-axis scale of the figure, nested test set accuracies for all learning approaches are at or near ceiling. The boxplots shown represent the median (line inside the box), first and third quartiles (bottom and top of the box, or the lower and upper hinges, respectively), and minimum and maximum values (lower and upper whiskers, respectively, or inner fences). Outliers exceeding 1.5 times the box height are shown as individual sample points (circles). Extreme outliers, or those samples exceeding 3 times the box height, are indicated by asterisks.

Implementing the k-fold (*k* = 10) procedure for each participant (using S1 as the learning set and S2 as the test set) for each of the *N* = 20 participants resulted in 200 trained/tested classifiers for each of the four learning approaches (LDA, SVM-LIN, SVM-RBF, and ANN). A modified analysis design from that used for the AF-MATB performance and NASA-TLX subjective workload data is necessary given that (1) the within-subject factor of workload is collapsed into a single algorithm performance metric, either proportion of epochs correctly classified (“accuracy”) or d', and (2) the within-subjects factor of session is eliminated given the desire to only investigate the simulated real-time implementation of the pBCI architecture performance on S2. Classifier performance on the nested test set (random 10% of S1) was at ceiling for all of the learning approaches (Figure 12) and is omitted from all further analyses. For all statistical tests the learning algorithm performance measure used was d'; however, to aid in ease of interpretation, all figures will present overall classifier accuracy as proportion of all epochs that were correctly classified.

**Figure 13:**
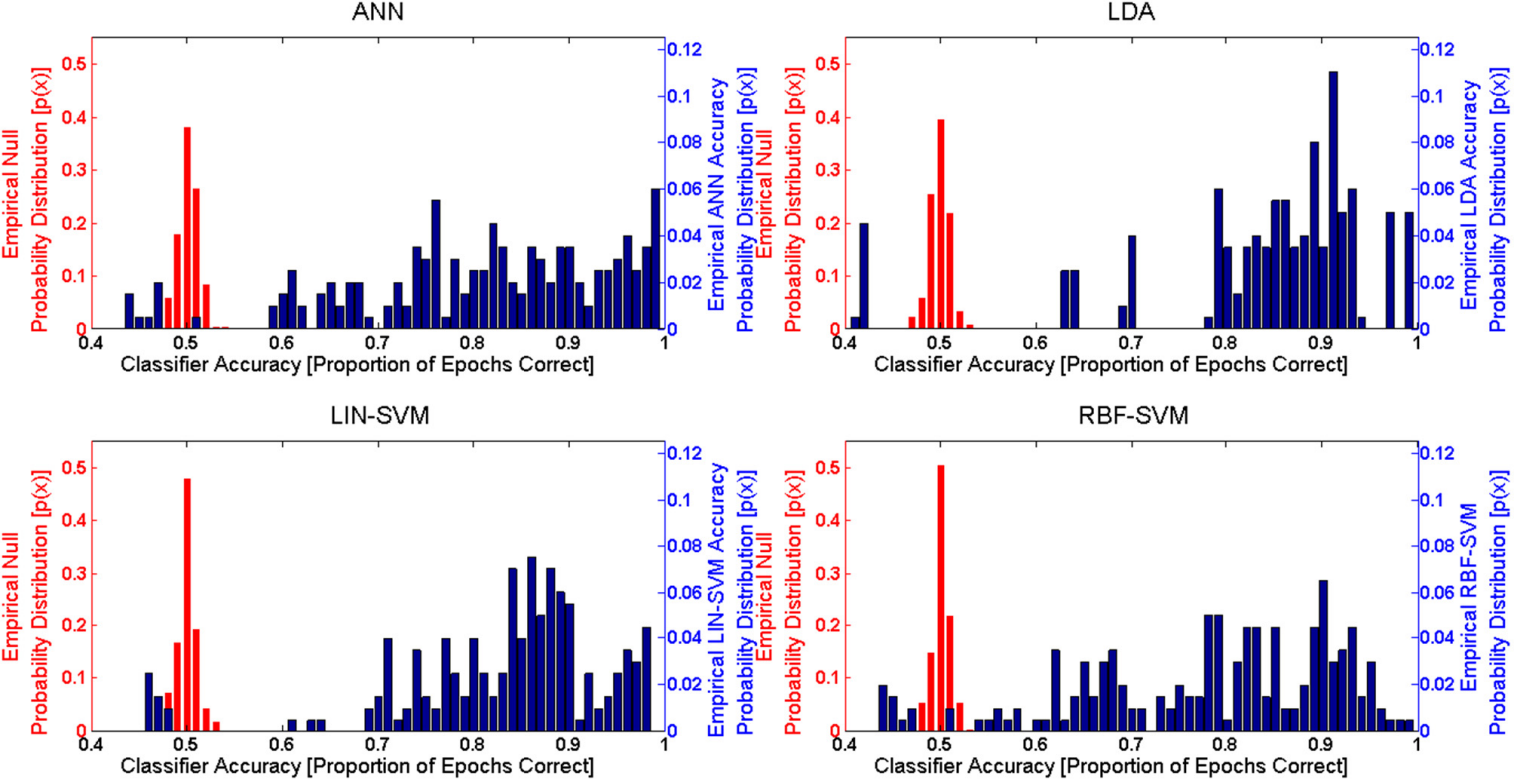
**Empirical probability distribution functions for both the null (left y-axis, in red) and between-session (right y-axis, in blue) classifier accuracies for each of the four learning approaches**. These empirical probability distribution functions are derived from the k-Fold procedure (*k* = 10) for both the empirical and between-session (S2) test sets. Note that the left y-axis (for null) and right y-axis (between-session) scales are different due to the varying accuracy probability ranges of the two distributions. The empirical null distributions, in the case of all learning approaches, are tightly bounded around the theoretical empirical null accuracy, 0.5 (50% random chance accuracy in the binary problem). Empirical between-session accuracies are significantly different (*p* < 0.01) from the empirical null accuracies for all learning approaches.

In order to evaluate learning algorithm performance, observed classifier performance was compared to the null distribution for each approach. Given the binary classification problem presented here, the theoretical null accuracy should be 0.50 (or 50% accuracy, with a theoretical null d' of 0). An empirical comparison requires that the empirical null distribution for classifier performance be available. Following the methods of Hughes et al. (2013), empirical null distributions were calculated for each of the learning approaches by randomizing class label assignments (while keeping the sets balanced) for both the learning and test sets. These empirical null distributions were determined via the same k-fold procedure used for the actual accuracy results. The learning data included in each of the empirical null k-folds was identical to that included in a corresponding accuracy k-fold (that is, the same exact same feature input matrices used for the accuracy k-folds were also used for the empirical null k-folds). Accuracy distributions from both k-fold procedures are shown in Figure 13.

The accuracy distribution (using d') for each individual learning approach was compared to its corresponding empirical null distribution using a paired *t*-test (two-tailed, α = 0.01) and the Bonferroni correction for multiple comparisons. In this series of analyses, each participant contributed a single median calculated across the folds for each approach. This choice was made for two reasons, first so that the sample size for learning algorithm performance is not inflated from that used in other analyses, and second so that spurious algorithm performance cases (if present) would not appear in the dataset as extreme values or outliers. All four learning approaches generated performance results that were significantly greater than their corresponding empirical nulls. These test results are summarized in Table 2.

**Table 2:** **Results of pairwise comparison tests for individual learning approaches compared to their respective empirical null distribution**.

**Learning approach**	***t*(19)**	***p*-value**	**μ**	**σ**	**99% CI**
ANN	8.6091	<0.001	2.3993	1.2464	[3.1967, 1.6020]
LDA	9.3152	<0.001	2.4695	1.1856	[3.2279, 1.7110]
LIN-SVM	9.3152	<0.001	2.4044	1.0390	[3.0691, 1.7398]
RBF-SVM	8.2681	<0.001	2.1190	1.1461	[2.8522, 1.3858]

A 2 (electrode replacement, between) × 4 (learning approach, within) mixed model ANOVA was performed to test for significant effects of these factors on learning algorithm performance. As with the empirical null comparisons, and with the same justifications, median classifier performance values were used in this analysis.

**Figure 14:**
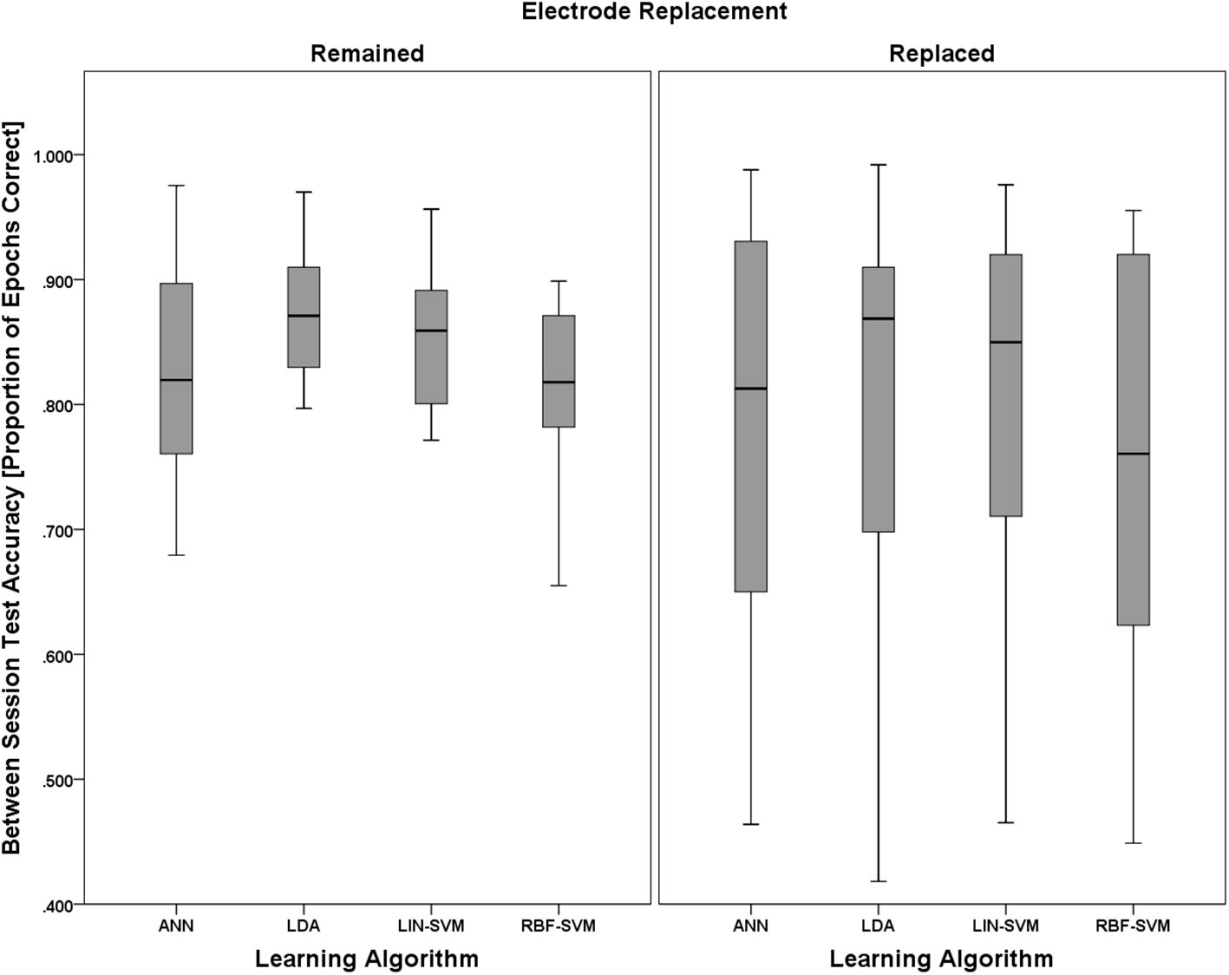
**Boxplots showing between-session classifier accuracy data representative of the 2 (electrode replacement, between) × 4 (learning approach, within) mixed model ANOVA**. The between-subjects factor of electrode replacement was not significant. The effect of learning approach was significant and is probed further in Figure 15. The boxplots shown represent the median (line inside the box), first and third quartiles (bottom and top of the box, or the lower and upper hinges, respectively), and minimum and maximum values (lower and upper whiskers, respectively, or inner fences). Outliers exceeding 1.5 times the box height are shown as individual sample points (circles). Extreme outliers, or those samples exceeding 3 times the box height, are indicated by asterisks.

Mauchly's test revealed no violations of sphericity, χ^2^_(5)_ = 6.542, *p* = 0.258; therefore, exact degrees of freedom were used in the following analyses. The main effect of electrode replacement was not significant, *F*_(1, 18)_ = 0.086, *p* = 0.773, η^2^_*p*_ = 0.005. There was, however, a significant main effect of learning approach, *F*_(3, 54)_ = 4.489, *p* = 0.007, η^2^_*p*_ = 0.200. The two-way interaction of (electrode replacement × learning approach) was not significant, *F*_(1, 18)_ = 2.593, *p* = 0.125, η^2^_*p*_ = 0.126. Boxplots for learning algorithm performance are shown in Figure 14.

**Figure 15:**
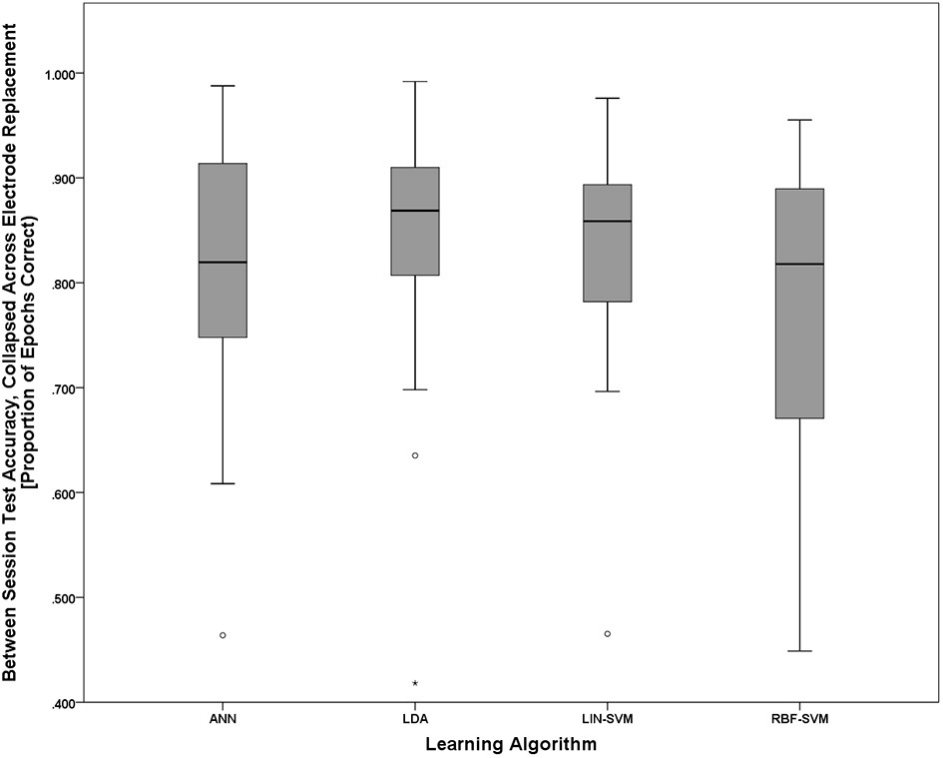
**Boxplots showing between-session classifier accuracy, collapsed across group, data representative of the *post-hoc* pairwise comparison testing given the significant main effect of learning approach shown in Figure 14**. Despite the significant main effect shown in Figure 14, all *post-hoc* pairwise comparison tests were non-significant (using the Bonferroni correction for multiple comparisons), although the two comparisons that approached significance were ANN vs. RBF-SVM and LDA vs. RBF-SVM. The boxplots shown represent the median (line inside the box), first and third quartiles (bottom and top of the box, or the lower and upper hinges, respectively), and minimum and maximum values (lower and upper whiskers, respectively, or inner fences). Outliers exceeding 1.5 times the box height are shown as individual sample points (circles). Extreme outliers, or those samples exceeding 3 times the box height, are indicated by asterisks.

To further probe the main effect of learning approach, a *post-hoc* pairwise comparison employing Bonferroni correction for multiple comparisons was performed on the learning approach factor (collapsed across electrode replacement), α = 0.01. The pairwise comparison tests revealed a difference of 0.282, 99% CI [−0.21, 0.586], that approached significance, *p* = 0.018, between ANN (*M* = 2.406, *SD* = 1.248) and RBF-SVM (*M* = 2.124, *SD* = 1.146) classifier performance. There was also a difference of 0.343, 99% CI [−0.085, 0.769], that approached significance, *p* = 0.05, between LDA (*M* = 2.467, *SD* = 1.178) and RBF-SVM (*M* = 2.124, *SD* = 1.146) classifier performance. All other comparisons were non-significant (*p* > 0.13). A boxplot outlining the *post-hoc* tests (collapsed across electrode replacement) is shown in Figure 15.

### Additional learning algorithm performance analysis

To test for cross-session generalization, all learning algorithms were trained on data from S2 and tested on S1 for between-session accuracy; all subsequent preparation of the learning algorithm performance results, expressed as d', was consistent with previous analysis where the learning set was extracted from S1 and the between-session test set consisted of all data from S2. A paired-samples *t*-test (two-tailed) was independently performed for each learning algorithm using the median learning algorithm performance from the k-fold distributions. The paired-samples *t*-tests, α = 0.01, were all non-significant, with a mean difference of 0.123, 99% CI [−0.200, 0.445], *p* = 0.291 for the ANN, 0.120, 99% CI [−0.174, 0.141], *p* = 0.257 for the LDA, 0.128, 99% CI [−0.214, 0.471], *p* = 0.297 for the LIN-SVM, and 0.030, 99% CI [−0.357, 0.418], *p* = 0.825 for the RBF-SVM. With all results being non-significant, a correction for multiple comparisons was not necessary. These results demonstrate very good cross-session generalization when using either session, S1 or S2, for the learning set.

While not the focus of this study, the *post-hoc* nature by which the dataset may be examined provides the opportunity for a number of additional, and informative, analyses. In particular, new learning approaches can be simulated using features that are derived from individual data sources, such as separating feature sets into those originating from EEG channels and those originating from non-EEG (or, peripheral) channels. Each of these new feature sets can also be tested for cross-session generalization (learning on S1 as compared to learning on S2). To this point, a number of different learning trials were performed by considering a variety of situations under which only certain classes (or sources) of features would be available. Each of these new feature sets was also tested for cross-session generalization. Tables containing learning algorithm performance metrics, separated for each participant and each group, can be found in the online Supplementary Material for this manuscript. Researchers interested in obtaining a copy of this dataset for additional analysis should contact the corresponding author.

As an example of an additional analysis that could be performed using these data tables, learning algorithm performance using the complete feature set (Figure 14) was compared to using only those features derived from EEG data channels. A paired-samples *t*-test (two-tailed) was independently performed for each learning algorithm using the median learning algorithm performance from the k-fold distributions. The paired-samples *t*-tests, α = 0.01, with a mean difference of 0.565, 99% CI [0.017, 1.112], *p* = 0.008 for the ANN, 0.437, 99% CI [0.151, 0.722], *p* = 0.0003 for the LDA, 0.488, 99% CI [0.083, 0.892], *p* = 0.003 for the LIN-SVM, and 0.372, 99% CI [−0.111, 0.857], *p* = 0.040 for the RBF-SVM, all revealed significant differences or approached significance between the two approaches without any correction for multiple comparisons (noting that only the LDA result remained significant, *p* = 0.0012, after Bonferroni correction, with the ANN, *p* = 0.032, and the LIN-SVM, *p* = 0.012, approaching significance; the RBF-SVM, *p* = 0.16, would be considered not significant). In each case, overall mean classifier performance for the group (expressed as sensitivity, or d') was higher using the complete feature set than using only EEG-derived features.

### Feature saliency and rankings for ANN learning set

Feature saliency was calculated from the ANN learning procedure using the Ruck saliency method (Ruck et al., 1990). Saliency values were converted to proportion of summed saliency (across all 37-features) for each of the 200 k-fold iterations and then averaged together to form an omnibus saliency ranking. The top five features, ranked by mean saliency, are shown in Table 3 (the full ranking table can be found in Supplementary Materials).

**Table 3:** **Mean saliency rank of features (top 5 of 37)**.

**Feature**	**Mean saliency rank**
Blink Rate	0.066357
O2 Gamma	0.043579
VEOG Gamma	0.034536
IBI	0.033598
P7 Gamma	0.033462

A second approach to examining feature saliency is to compare the ordinal rank of the features, regardless of their relative saliency, within a single learning iteration. This results in a very simple measure, the average rank (range of 1–37) for each of the features used in the learning set. This average rank measure was also computed across the 200 k-fold iterations (*N* = 20, *k* = 10) for the ANN learning approach. Results for the top five features, ranked by mean ordinal position, are presented in Table 4 (the full ranking table can be found in Supplementary Materials).

**Table 4:** **Mean ordinal rank of features (top 5 of 37)**.

**Feature**	**Mean ordinal rank**
IBI	11.65
P7 Alpha	17.32
O2 Beta	17.95
Fz Theta	18.18
Pz Beta	18.445

## Discussion

The study presented here aimed to investigate the impact of methodological variability due to same-day, between-session electrode replacement on learning algorithm performance in the context of a pBCI system for assessing cognitive workload. The importance of understanding these effects can be easily understood when considering that (1) real-world implementation of pBCI systems will almost necessarily be implemented for multi-session and multi-day use, and (2) sensor systems for monitoring neurophysiological and neurobehavioral measures in these architectures will almost necessarily require removal and replacement between sessions. Decoupling this effect from previously observed declines in classifier performance over time courses as short as hours (Christensen et al., 2012) necessitated a between-subjects design to probe electrode replacement as a factor. This prior observation, when considered in tandem with the logistical difficulty of maintaining electrode montage preparation in a non-clinical setting for extended time periods, made the selection of a time course of minutes to hours the logical choice over which to investigate these effects.

A critical first-step analysis was to observe electrode impedance for the duration of the data collection. Neither the between-subjects factor of electrode replacement nor the within-subjects factor of time of measurement significantly impacted the omnibus measure of electrode impedance. This result strongly suggests that any electromechanical variability introduced by both a second electrode preparation and the use of different electrode sets between sessions did not influence data quality (either negatively or positively) for the Replaced group. A second conclusion that can be drawn is that electrode impedances were also maintained at acceptable levels for the Remained group.

There were two participants who, at the beginning of S2 (impedance measurement point Z3 in Figure 2), had one or more electrodes with impedances above set tolerance maximums (one electrode for the first participant, and four electrodes for the second participant), all of which were on electrodes at EEG scalp sites. In each instance, adding more Electro-Gel to the site reduced the impedance to within tolerance; additional skin preparation was not required. Data quality was not noticed to be affected by any impedance changes that may have occurred during S1 (all raw and real-time processed time series were viewed online during data collection). The effect of the addition of Electro-Gel, when coupled with observations of researchers during data collection, indicates that the conductive gel leaked from underneath the plastic housing on the electrode cap during the between-session break. Out-of-tolerance impedances were never reported for either the single-lead (VEOG, HEOG, and mastoid) or disposable electrodes (ECG). Both of these electrode types seal to the skin via temporary adhesive and necessarily prevent gel leakage, whereas the plastic housings on the elastic electrode cap are more easily separated from the skin surface under some conditions (i.e., inadvertent displacement, less-than-perfect conformity of the cap to the participant's head, varying hair styles, etc.).

Also worth noting is the potential for variability in electrode location due to replacement between sessions. All electrode preparations for this study were completed by experienced EEG researchers; as such, it is reasonable to assume that electrode location was consistent for the Replaced group. Even small variations in physical electrode locations are likely to be negligible given the volume conduction phenomenon in skin surface electropotential measurement, which can further be interpreted as the cause of the often reported poor (native) spatial resolution of EEG (Gevins, 1987).

The dual-session approach is a potential confound in this study design. Workload may vary between sessions due to participant task learning or fatigue, resulting in degraded pBCI performance. However, results obtained with subjective workload and task performance measures suggest that workload was highly consistent across sessions. The only significant effect observed for both task performance and subjective workload was that of task difficulty. These complementary results show that the manipulation of task difficulty was successful in influencing cognitive workload, or more precisely, increased workload between low and high task difficulty (as evidenced by the subjective measures) such that task performance decreased (as evidenced by the task performance measures). The consistency of these measures across sessions confirms that workload state, respective of task difficulty, can be considered constant for both S1 and S2. It is also evident that both the Remained and Replaced groups experienced the same relative workload levels.

With all of the aforementioned variables being equal with respect to electrode replacement and session, and a meaningful difference in workload evidenced between task difficulty conditions, it is thus appropriate to make comparisons in learning algorithm performance given a pBCI system approach for assessing cognitive workload. Accuracy distributions for all learning approaches, compared to their respective empirical null distributions, showed significant performance above the chance accuracy level. While *post-hoc* comparisons between learning algorithms did not reach significance, there is evidence to suggest some differences in performance between the algorithms used. Considering the nested test set (reserved from S1) accuracies in Figure 12 together with the between-session (tested on S2) accuracies in Figure 15, it appears that both the LDA and LIN-SVM learning techniques produced slightly better generalization to S2 (between-sessions) at the cost of lower overall nested test set accuracy, suggesting the possibility of over-fitting in the non-linear approaches. Indeed, the RBF-SVM exhibited very high nested test set accuracies only to perform worst, overall, when fixed as a pattern classifier for testing on S2. The ANN, with its early stopping rule based on learning error from a withheld validation set, appears to strike a balance between robust learning and over-fitting. It is worth noting that the validation set is not strictly independent from the learning set since they were both sampled from S1. A more thorough methodology would be to use a validation set with greater independence from the learning set, such as data from a third session, or even perhaps a different day. Given this consideration the ANN still showed robust generalization to the between-session test set while maintaining nearly perfect nested test set accuracy.

Overall learning algorithm accuracies presented here, as related to temporal distance between learning and test sets, largely replicate those obtained using a very similar cognitive workload task in previous work (Christensen et al., 2012). Namely, we observed workload state classification for data temporally separated from the learning set by only seconds to perform at or near ceiling (Figure 12). Further, classification accuracy for data temporally separated from the learning set (S1) by minutes to hours (S2) suffers from a decrement in accuracy relative to the nested test set from the same session (Figure 15). It is noteworthy to state, here, that the temporal delay between S1 and S2 was 45 min, which is comparable to the “minutes” of separation category in Christensen et al. (2012). At this level of separation from learning to test, both studies produced classification accuracies of 85–90%, on average.

The most important result of this work, however, is that learning accuracy was not impacted by the replacement of the electrode montage between sessions. The impact of this finding is perhaps even greater considering that a new set of electrodes was applied in between sessions for the Replaced group. Eliminating this methodological variability as a potential factor in learning algorithm performance is a key step forward in developing strategies for implementing multi-session, multi-day paradigms for pBCI usage. While only one feature set was tested here, it is reasonable to believe that similar results would also be obtained for evolving signal processing methodologies that are being actively developed and used elsewhere (see Makeig et al., 2012 for a recent review). It is also reasonable to hypothesize that this result would also transfer to other task protocols given that the electrode preparation is uniquely independent from the underlying cognitive task protocol; however, it is important for future work to consider the expansion of these considerations in regard to other protocols as well, such as steady-state conditions of shorter duration than those used here (15 min task states), dynamic, and concurrent task states. Additional analyses of learning algorithm performance showed good generalization of these results when using S2 as the learning set and S1 as the test set. Also of interest is that the addition of the perhipheral physiological measures to the feature set increased overall classifier performance for all four learning approaches, with only the RBF-SVM not approaching or obtaining significance as compared to using the EEG-only feature set.

The feature saliency analysis revealed similarities and differences between this study and previously-published results regarding EEG signals associated with workload. For example, Wilson and Fisher (1995) reported significant contributions from higher frequency bands including gamma, while Gevins et al. (1998) reported increased frontal theta and decreased parietal alpha with increasing workload. Both patterns of results were found in this study, depending on which approach to determine feature saliency rank was used. Saliency-based assessment showed three of the top five features in the gamma band, while ordinal rank assessment showed three different bands (theta, alpha, and beta) from four different sites as being most highly ranked. Both included IBI, and Blink Rate was the most salient over all (on average). One reasonable interpretation for the difference in saliency vs. ordinal rankings is that features such as gamma band activity are very highly separable but may not appear frequently for all participants, while other features that are less separable (weaker learners) may be more consistently present across a group of participants. Additional evidence in favor of this interpretation is found in the analysis approaches taken in prior studies; Wilson and Fisher (1995) obtained their results implicating gamma activity via individually trained classifiers, while Gevins et al. (1998) analyzed data at the group level and found frontal theta and parietal alpha to be significant indicators of workload. This result suggests that the use of a diverse sensor suite and continued investigation of new sensor types and feature extraction techniques are worthwhile endeavors for those interested in pBCI system research. As an example, Whitham et al. (2007, 2008) have provided convincing evidence that beta and gamma bands are heavily influenced by tonic eletromyographic artifact (EMG); given the large amplitude of EMG activity, even when projected to scalp EEG sites, it is reasonable to infer that high-amplitude EMG differences associated with workload state changes could be responsible for highly-separable beta and gamma band features. If it is the case that EMG activity happens to be a useful “feature” for some pBCI applications, a systematic investigation of this effect in the context of cognitive and affective state assessment that leverages relevant feature separation and extraction approaches (e.g., McMenamin et al., 2010, 2011) would be a worthwhile effort.

There are a number of reports from researchers suggesting less-than-perfect success rates, or the so called “BCI-illiterate” effect, in traditional BCI applications (e.g., Guger et al., 2009, 2011; Allison et al., 2010), so it is not at all surprising that pBCI architectures can produce low-performing state classification accuracies for some participants. Of the 20 participants in this study, one (de-identified with an identifier of P24) was consistently at or below chance accuracy on between-session test set accuracy; this below chance accuracy persisted when S2 was used as the learning set, as well. This participant's low-performing workload state classification impacted the sample distributions shown in Figure 14 by negatively skewing the learning algorithm performance of the electrodes replaced group. Given a lack of any a prioi basis on which to exclude these results, P24's data was including in all prior analysis; however, given the significant skew, it is worthwhile to investigate learning algorithm results without this participant's data. In order to examine the learning approach data in such a way, the skewed data from P24 was replaced with the sample mean (by factor) and the distributions were reexamined. These data, along with corresponding time series data similar to that shown in Figure 11, are included in the online Supplementary Material (Figures 2–4). Unsurprisingly, replacing P24's data with the sample mean (by factor) all but eliminates the skew from the data distributions (Supplementary Material, Figure 2). As a comparison, this same data is also expressed as d', the learning algorithm performance measure that was used for all statistical analysis (Supplementary Material, Figure 3). As with the classifier accuracy representation, there is no noticeable skew represented in the learning algorithm performance distributions when expressed as d'; note, also, that the normality and equality of variance across factors is greatly improved in the d' distributions, thus further justifying the use of d' as a suitable metric for all analyses of variance. Repeating the previously reported 2 (electrode replacement, between) × 4 (learning approach, within) mixed model ANOVA to test for significant effects of these factors on learning algorithm performance after correcting for P24 as an outlier produces nearly identical results: using exact degrees of freedom (no violation of sphericity via Mauchly's test, χ^2^_(5)_ = 6.560, *p* = 0.256), the main effect for electrode replacement was not significant, *F*_(1, 18)_ = 0.084, *p* = 0.775, η^2^_*p*_ = 0.005. There was, however, a significant main effect of learning approach, *F*_(3, 54)_ = 5.131, *p* = 0.003, η^2^_*p*_ = 0.222. The two-way interaction of (electrode replacement × learning approach) was not significant, *F*_(1, 18)_ = 0.789, *p* = 0.505, η^2^_*p*_ = 0.042. That is to say that correcting for P24 as an outlier participant does not affect the outcome of the test for learning approach performance. When compared to Figure 11, the time series data for this participant (Supplementary Material, Figure 4), does not exhibit any easily identifiable features that appear to be separable with respect to changes in workload. An understanding of why pBCI systems may work for some persons but not others (or at least may not be as accurate) could be tremendously helpful, enabling adaptations in sensor choice, feature selection, training procedures, and other such interventions to mitigate those differences.

In summary, this work set out to determine what, if any, impact electrode removal and replacement has on learning algorithm performance in dual-session, same-day use of pBCI systems. Testing was conducted over a time course of minutes to hours, known from prior work to result in observable declines in algorithm accuracy comparable with those observed over multi-day testing. Results showed that, after successfully implementing a paradigm for increasing cognitive workload in a multitask environment, the accuracy for a group of participants whose electrodes were replaced in a between-session test did not significantly differ from a control group whose electrodes remained in place for the entire data collection. Having reduced concern for this potential source of methodological variability as a confound to learning accuracy decline in dual-session paradigm, it is recommended that future work in this area focus on non-stationarity and reduced classifier performance due to intrinsic factors not related to the removal and replacement of electrodes. However, it is also pertinent that this type of study be repeated and replicated in other paradigms for increased validity of the results presented here. Future pBCI research should also strongly consider novel sensor and feature development in an effort to improve the long-term stability of these systems, particularly for real-world applications (e.g., McDowell et al., 2013).

### Conflict of interest statement

The authors declare that the research was conducted in the absence of any commercial or financial relationships that could be construed as a potential conflict of interest.
