# Supplementary material for: Electrode replacement does not affect classification accuracy in dual-session use of a passive brain-computer interface for assessing cognitive workload
Source: Front Neurosci. 2015 Mar 9;9:54. doi: 10.3389/fnins.2015.00054 (PMC4353251; doi:10.3389/fnins.2015.00054)
Supplement: Supplementary file 1 [file DataSheet1.PDF]

Supplementary Material

Electrode replacement does not affect classification accuracy in multi-session use of a passive brain-computer interface for assessing cognitive workload

Justin R. Estepp[1]*, James C. Christensen[1]

[1]Applied Neuroscience Branch, Human Effectiveness Directorate, 711th Human Performance Wing, Air Force Research Laboratory, Wright-Patterson AFB, OH, USA

*** Correspondence:** Justin R. Estepp, [1]Applied Neuroscience Branch, Human Effectiveness Directorate, 711th Human Performance Wing, Air Force Research Laboratory, 2510 Fifth Street, Wright-Patterson AFB, OH, 45433, USA.

Justin.estepp@us.af.mil

1. AF-MATB Simulation Environment

Additional information regarding the Air Force Multi-Attribute Task Battery (AF-MATB; Miller, 2010) implementation and the task training process are presented here as supplementary materials. This information is useful for a more comprehensive understanding of AF-MATB task parameters and operation, as well as the extensive, five-day training process that was used to mitigate against task learning effects and identify individual high task difficulty levels for each participant.

AF-MATB, designed to be broadly representative of aircraft operation, is comprised of four unique subtasks that can be simultaneously operated in any combination. These four subtasks consist of compensatory manual tracking (Tracking), visual stimulus monitoring (System Monitoring), auditory stimulus monitoring (Communications), and dynamic resource allocation (Resource Management) tasks. For this study, all four subtasks were operated concurrently by the participants. In addition to the four subtasks, which appear on the interface as shown in Figure 1, the AF-MATB simulation interface contains two additional panels: the Pump Status panel and the Scheduling panel. The Pump Status panel contains the instantaneous flow rates (in arbitrary units) for the eight individual pumps in the Resource Management subtask and can be used by the participants to easily identify current pump states (described in more detail in section 1.1.4). The Scheduling panel can be used to convey information about future simulation state of the Tracking and Communications subtasks to participants but was disabled for this study (and appeared to participants as shown in Figure 1).

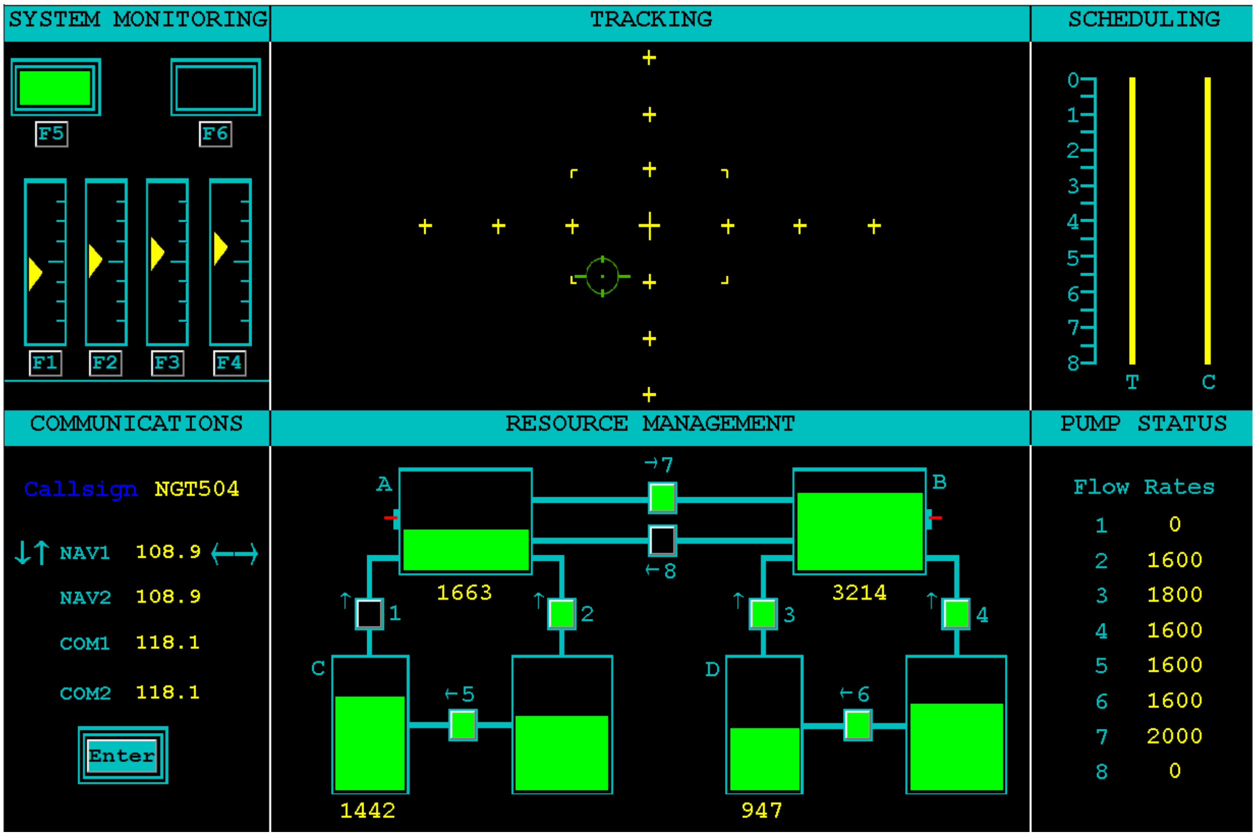

Figure 1 User interface for the AF-MATB task environment. The four subtasks (System Monitoring, Tracking, Communications, and Resource Management) are shown in the left and center columns on the interface. The right column shows Scheduling and Pump Status information windows. The Scheduling information window was disabled for this study. More information on the AF-MATB task can be found in AF-MATB User's Guide (Miller, 2010).

The AF-MATB simulation environment is highly customizable and flexible, and the number of modes and adjustable parameters available to a researcher or practitioner are beyond the scope of the work presented here. Interested readers may consult the AF-MATB User's Guide (Miller, 2010) or contact the corresponding author of this manuscript for the most up to date version of both the task software and documentation (which are freely available for distribution and use). The task parameters and settings used for this study were chosen in order to replicate the original MATB task functionality (Comstock and Arnegard, 1992).

1.1.1. Tracking

The tracking subtask requires participants to compensate for random movement of the reticle (in green) in order to keep it centered on the target crosshairs (in yellow). Random movement of the reticle is governed by drift velocity and perturbed at irregular time intervals by changes in direction; these two parameters (drift velocity and frequency of directional changes) are used to influence compensatory tracking difficulty. The reticle is controlled by the participants with a joystick. Difficulty of the tracking subtask is increased over three levels (low, medium, and high) by which the drift velocity and frequency of directional perturbations are increased. Performance on the Tracking subtask is commonly expressed as a measure of distance from reticle to the target, such as root-mean-squared (RMS) error, for the duration of the simulation.

1.1.2. System Monitoring

The System Monitoring subtask is divided into two separate visual stimulus types, lights and gauges. Both are identical in their operation; when any of the System Monitoring indicators show a warning state, the participant must respond via key press to reset the indicator. A warning state for the lights is either the green light (on the left, currently shown as 'on' in Figure 1) turning off or the red light (on the right, currently shown as 'off' in Figure 1) turning on. The gauges, which slowly oscillate about the center tick mark, enter a warning state when they drift outside one tick mark above or below center. All indicators in Figure 1 show their default state. The participant responds to a warning indicator by pressing its corresponding function key (F1 through F6, as shown in Figure 1) on the task keyboard; correct warning indicator acknowledgments are scored as a 'hit'. Any warning indicator not acknowledged by the participant within a predetermined time window will reset to its default state and be scored as a 'miss'. If a key press for any indicator is recorded while that indicator is in its default state, that acknowledgement is scored as a 'false alarm'. Difficulty of the System Monitoring subtask is increased by increasing the frequency of warning indicators. Performance on the System Monitoring subtask is commonly expressed as the percentage of warning indicators scored as 'hits'.

1.1.3. Communications

The Communications subtask requires the participant to monitor short voice communications (voice comms) played over a set of desktop speakers that contain three pieces of information: a callsign, a communications channel, and a frequency. The participant is assigned a callsign (NGT504) and is instructed to listen for voice comms designated for his or her callsign; these are labeled as 'target' voice comm events. Some voice comms are designated for distractor callsigns, labeled as distractor events, and should be ignored by the participant. If identified as the participant's callsign, the participant must follow instructions to change one of four communication channels (NAV1, NAV2, COM1, or COM2) to a specified frequency. This is accomplished via the arrow keys (up and down to change the channel, left and right to change the frequency) on the task keyboard. All channel/frequency modifications must be confirmed by pressing the 'Enter' button on the task keyboard. 'Hits' in the Communications subtask must be correct in callsign, communications channel, and frequency, and confirmed by pressing the 'Enter' button. If any of these are incorrect, or if the voice comm is not acknowledged by the participant within a predefined time window, the voice comm event will be counted as a 'miss'. Voice comm confirmations (pressing the 'Enter' key) that are designated for a distractor callsign or fall outside of a predefined response window, even if correct, were counted as a 'false alarm'. Difficulty of the Communications subtask is increased by increasing both the frequency of target voice comms and the ratio of target to distractor voice comm events. Performance on the Communications subtask is commonly expressed as the percentage of voice comms scored as 'hits'.

1.1.4. Resource Management

The Resource Management subtask focuses on two simulated fuel tanks (designated as Tank A and Tank B in Figure 1). Each tank begins at a nominal fuel level (indicated by the red tick mark on each tank, a volume of 2500 arbitrary fuel units), but the fuel tanks deplete at constant rates during the simulation. The participant is required to keep each tank as close to its nominal fuel level as possible by resupplying from four reserve tanks (designated as Tank C, Tank D, and two unlabeled tanks in Figure 1). The two unlabeled tanks hold an infinite amount of reserve fuel, but Tanks C and D are limited to a maximum capacity of 2000 fuel units. A series of fuel pumps, labeled 1 through 8, are used to control fuel flow between the tanks, and the direction of flow is indicated by an arrow next to

each pump. Instantaneous flow rates of all pumps are displayed in the Pump Status panel. A flow rate of '0' indicates that the pump is currently in the closed position (Pump 1 in Figure 1, shown with a graphical indicator of the pump being black), and any flow rate greater than 0 indicates that the pump is currently in the open position (Pump 2 in Figure 1, shown with a graphical indicator of the pump being green). Fuel pumps are cycled through the open and closed positions by pressing the corresponding pump number on the number key row.

There are two types of fuel pump events that can occur during a simulation: pump shut-offs and pump failures. A pump shut-off occurs when a pump switches from open to closed without any input from the participant. Any fuel pump that experiences a shut-off can be immediately opened again by the participant if he/she chooses to perform that action. Further, any pump that experiences a shut-off event while in the closed position stays closed (a pump shut-off cannot change a pump status from closed to open). A pump failure, however, completely disables a pump for a predetermined period of time regardless of previous pump status (open or closed). Pump failures are graphically indicated by turning the pump red and setting the flow rate to 0 in the Pump Status panel. Pump failures will resolve themselves after a period of time after which they will return to their normal functionality, beginning in the closed position. Difficulty of the Resource Management subtask is increased by increasing the frequency of both pump shut-offs and failures. Performance on the Resource Management subtask is commonly expressed as an average measure of deviation from the nominal fuel level in Tank A and Tank B, such as average RMS error for the two tanks.

1.2. AF-MATB Training and Titration: Days 1 through 5

To mitigate against task learning effects, participants were trained on AF-MATB for a minimum of 5 training sessions lasting 2 hours each (for a total of 10 hours of training). Training was guided by establishing 20 unique levels of task difficulty whereby the event frequencies for each subtask were increased (or, for Communications subtask distractors, increased and then decreased) step-wise between each level. These 20 levels and their assigned subtask event frequencies are shown in Table 1. Frequencies are expressed as the number of events in each subtask per 15 minutes of simulation time. The relationship between event frequency and task difficulty level is approximately linear, although minor variations between levels do exist. The level of Tracking difficulty for each level (low, medium, or high) is also indicated in Table 1.

Table 1 AF-MATB task difficulty levels and their associated subtask event counts in a 15-minute trial.

Level	Tracking	Communications		System Monitoring		Resource Management	
		Target	Distractor	Lights	Gauges	Failures	Shut-Offs
**1**	Low	9	3	72	63	6	3
**2**	Low	12	6	84	78	9	6
**3**	Low	15	9	96	93	12	6
**4**	Low	18	12	108	108	15	9
**5**	Medium	21	15	120	123	21	12
**6**	Medium	24	12	132	138	27	12
**7**	Medium	27	15	144	153	30	15
**8**	Medium	30	15	156	168	36	18
**9**	Medium	33	18	168	183	42	21
**10**	Medium	36	18	180	198	45	21

**11**	Medium	39	18	192	213	51	24
**12**	Medium	42	21	204	228	57	27
**13**	High	45	18	216	243	60	30
**14**	High	48	18	228	258	66	30
**15**	High	51	15	240	273	72	33
**16**	High	54	15	252	288	75	33
**17**	High	57	12	264	303	81	36
**18**	High	60	9	276	318	87	39
**19**	High	63	6	288	333	90	42
**20**	High	66	3	300	348	93	45

Task training was administered by a subject matter expert (SME) with extensive experience and familiarity with AF-MATB. On Day 1 of the five-day training protocol, participants were introduced to AF-MATB subtask concepts via written instruction provided by the SME. Participants were then given practice trials on each individual subtask during which they were required to demonstrate proficiency in understanding of the subtask functionality. During Days 2 and 3 the participants began performing all subtasks simultaneously, beginning at Level 2 and progressing upward at the discretion of the SME. Training scenarios lasted 5 minutes each, and a two hour session totaled between 10 and 15 trials with some replication of levels. The goal for the end of Day 3 was to identify a maximum task difficulty level at which the participant was able to achieve asymptotic task performance. As participants were instructed not to prioritize any one subtask over another, but to treat all subtasks as equally important, there was some variability in relative subtask performance between participants. Prior work in psychophysiological assessment of cognitive workload demonstrating the importance of individual differences in task ability suggests that asymptotic performance will vary between individuals (Wilson and Russell, 2007). As a result the selection of an individual participant's maximum/asymptotic task difficulty level, called their titration level, was estimated based on training performance through Day 3. Concurrence on estimated titration level was required between a minimum of two SMEs. To check the validity of the estimated titration level, each participant completed three, 5-minute trials each spanning two levels below and two levels above their estimated titration level on Day 4. Performance on these 15 trials completed on Day 4 was used to determine the participant's final titration level, which also required concurrence between a minimum of two SMEs. Finally, on Day 5, participants completed a minimum of two AF-MATB trials at their titration level, only these trials were extended to 15 minutes in length.

2. Full Saliency Rank and Ordinal Rank Feature Tables

'Top 5' feature tables for both saliency and ordinal rank features were presented in the manuscript. The full tables, including all 37 features, for both saliency rank (Table 2) and ordinal rank (Table 3) are presented here.

Table 2 Mean saliency rank of features.

Feature	Mean Saliency Rank
**Blink Rate**	0.066357
**O2 Gamma**	0.043579
**VEOG Gamma**	0.034536
**IBI**	0.033598
**T5 Gamma**	0.033462

**T5 Beta**	0.032797
**O2 Beta**	0.030498
**HEOG Delta**	0.029689
**Pz Gamma**	0.028665
**VEOG Alpha**	0.028326
**HEOG Gamma**	0.028051
**Fz Gamma**	0.027443
**F7 Gamma**	0.026488
**F7 Delta**	0.02626
**Fz Alpha**	0.026125
**T5 Delta**	0.025753
**Pz Beta**	0.025736
**Fz Beta**	0.025727
**HEOG Beta**	0.025122
**VEOG Theta**	0.024915
**Fz Delta**	0.024706
**O2 Theta**	0.024603
**T5 Alpha**	0.024569
**Pz Theta**	0.023866
**VEOG Beta**	0.023705
**F7 Theta**	0.023226
**F7 Beta**	0.023056
**Pz Alpha**	0.022999
**F7 Alpha**	0.022186
**HEOG Alpha**	0.022141
**Fz Theta**	0.021846
**Pz Delta**	0.02159
**O2 Delta**	0.020801
**T5 Theta**	0.020236
**HEOG Theta**	0.019183
**O2 Alpha**	0.019143
**VEOG Delta**	0.019017

Table 3 Mean ordinal rank of features.

Feature	Mean Saliency Rank
**IBI**	11.65
**T5 Alpha**	17.32
**O2 Beta**	17.95
**Fz Theta**	18.18
**Pz Beta**	18.445
**O2 Delta**	18.445
**O2 Alpha**	18.56
**VEOG Gamma**	18.59
**O2 Theta**	18.61
**T5 Beta**	18.615
**HEOG Theta**	18.67

**HEOG Alpha**	18.67
**T5 Gamma**	18.72
**Fz Gamma**	18.74
**T5 Delta**	18.765
**Pz Gamma**	18.885
**HEOG Gamma**	18.91
**VEOG Theta**	19.025
**VEOG Delta**	19.19
**Blink Rate**	19.24
**HEOG Delta**	19.255
**Pz Delta**	19.295
**O2 Gamma**	19.33
**HEOG Beta**	19.335
**Pz Theta**	19.355
**VEOG Beta**	19.405
**F7 Beta**	19.46
**F7 Theta**	19.465
**VEOG Alpha**	19.575
**T5 Theta**	19.73
**Pz Alpha**	19.925
**Fz Alpha**	20.16
**Fz Beta**	20.21
**F7 Delta**	20.33
**F7 Gamma**	20.715
**F7 Alpha**	20.965
**Fz Delta**	21.31

3. Data from Participant With Low-Performing Workload State Classification

As mentioned in the text, one participant consistently generated between-session classifier accuracies of approximately chance. The classifier accuracy distributions with this participant's data replaced with sample mean (by factor) are shown in Figure 2. The same data, only expressed as d', is shown in Figure 3. The time series data for this participant are shown below in Figure 4.

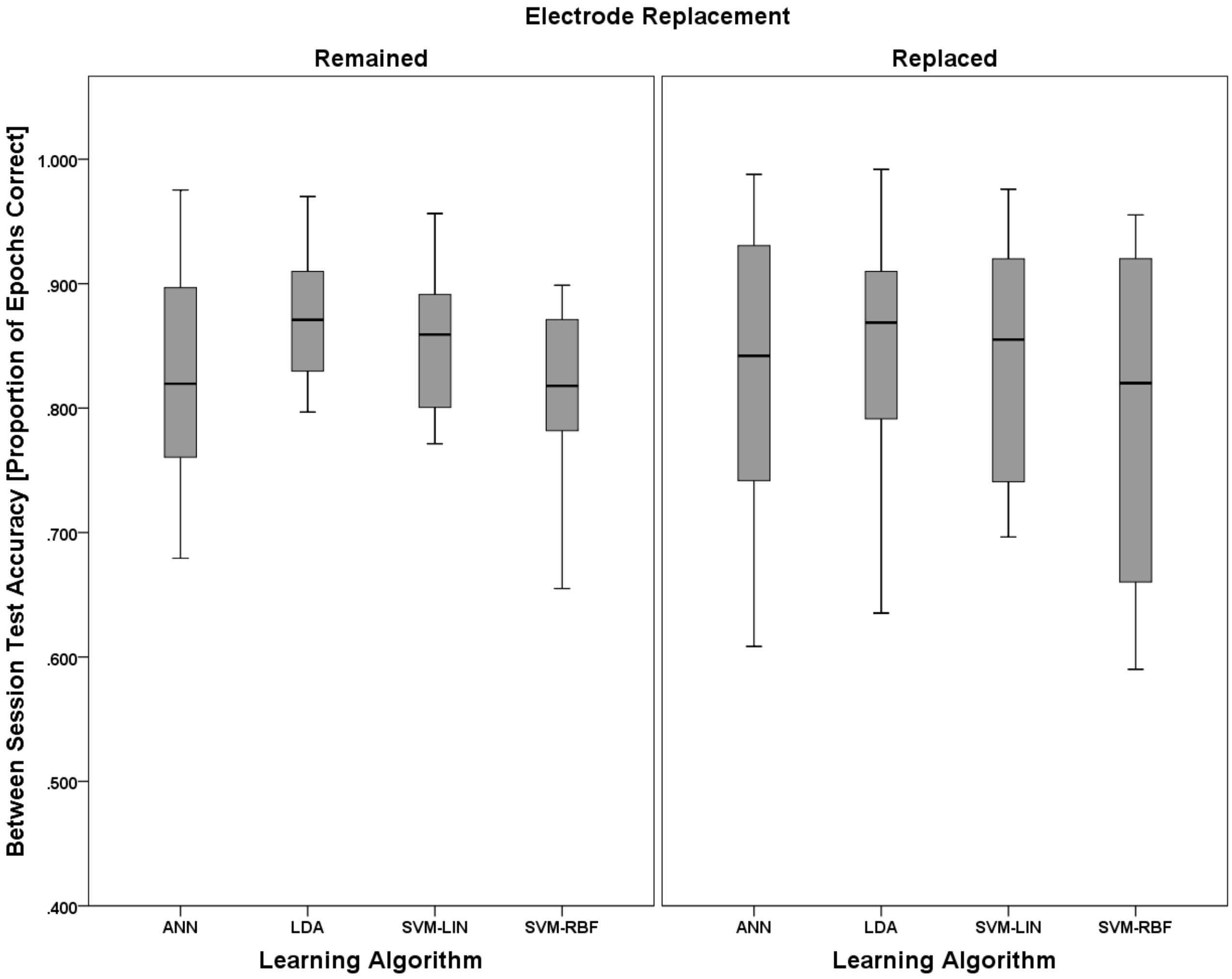

Figure 2 Boxplots showing between-session classifier accuracy data representative of the 2 (electrode replacement, between) x 4 (learning approach, within) mixed model ANOVA. This is the same data as shown in Figure 14 of the associated manuscript, only the participant (in the electrodes replaced group) with workload start accuracies at or below chance has been replaced with the sample mean (by factor). Note that the skew is all but eliminated when this participant's data is classified as an outlier and replaced with the sample mean (by factor).

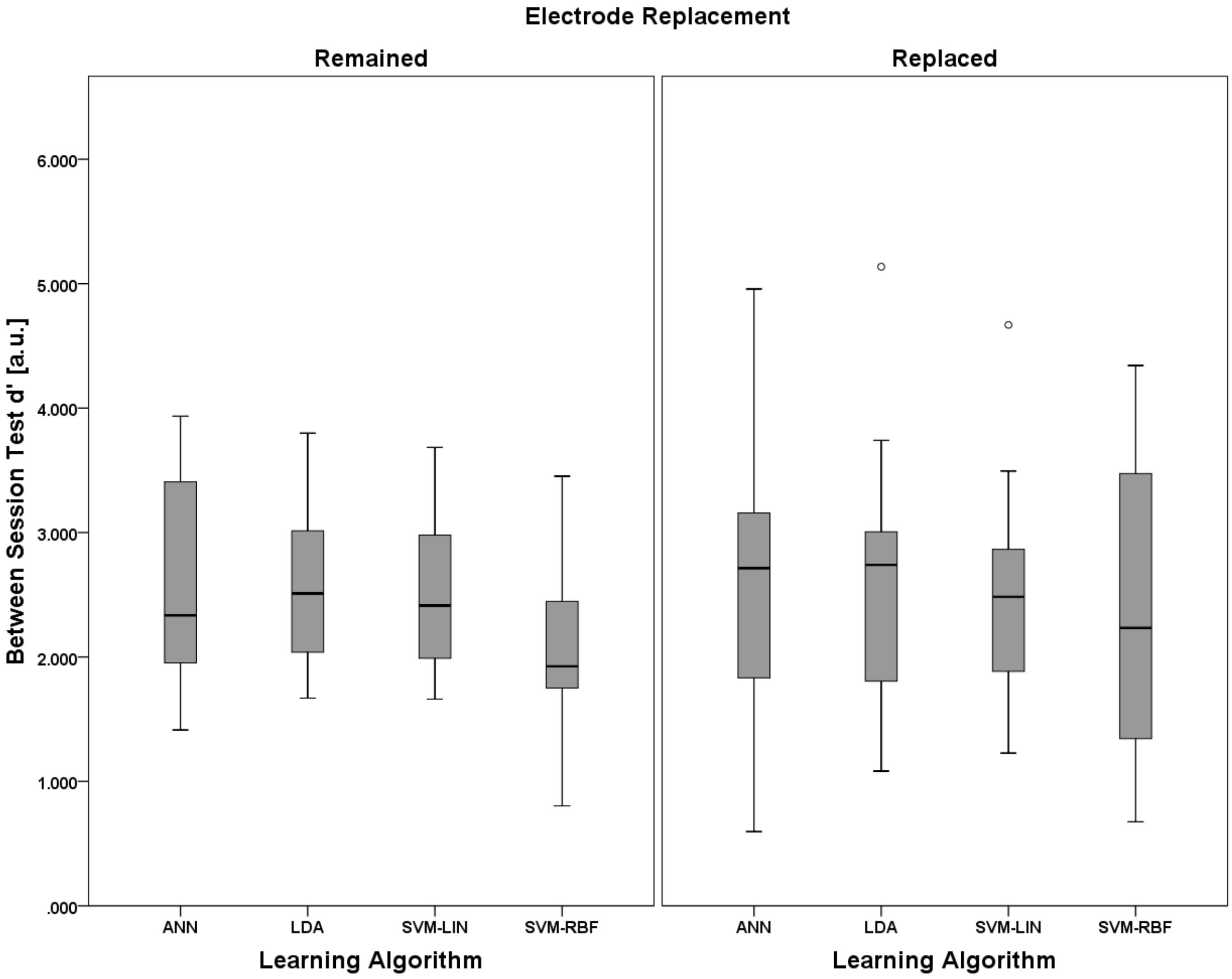

Figure 3 Boxplots showing between-session classifier d' data representative of the 2 (electrode replacement, between) x 4 (learning approach, within) mixed model ANOVA. This is the same data as shown in Figure 2 of this supplementary material, only expressed as d' (instead of classifier accuracy). As with Figure 2, the participant (in the electrodes replaced group) with workload start accuracies at or below chance has been replaced with the sample mean (by factor). As with Figure 2, the skew is all but eliminated when this participant's data is classified as an outlier and replaced with the sample mean (by factor). In addition, this figure, when compared to Figure 2, serves as an example of the improved normality and equality of variance for the data distributions when using d' instead of classifier accuracy as a measure of learning algorithm performance.

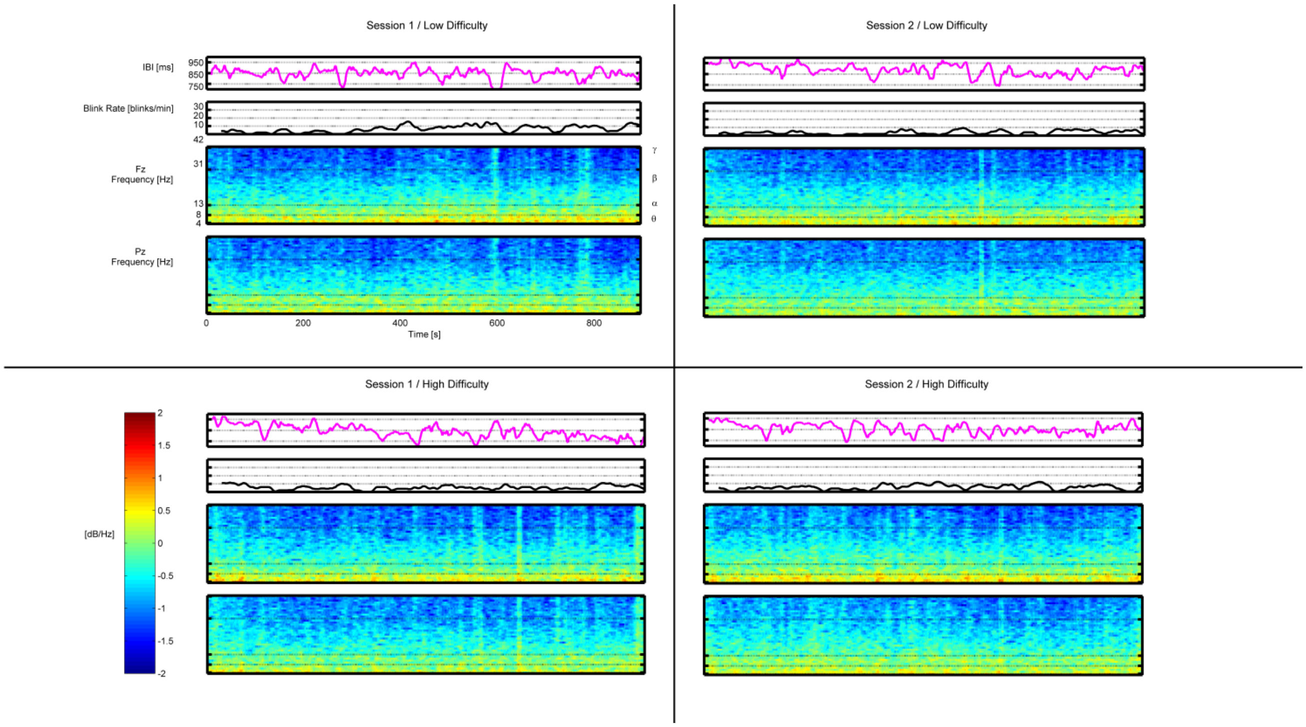

Figure 4 Feature plots for IBI, Blink Rate, Fz, and Pz (shown as time-frequency plots) for a participant at chance classifier accuracy. Feature plots for this participant are shown respective of task difficulty (in rows) and session (in columns). All corresponding feature plots are shown on the same y-axis scale (e.g., all time-frequency plots for Fz and Pz are shown using the scale depicted on the included colorbar). Frequency band ranges are shown on the time-frequency plots. In contrast to the participant presented in the manuscript, there are no clear features in the participant presented here that are clearly separable with respect to low versus high task difficulty. There are no visually noticeable effects of session, or as is the case for this participant, electrode replacement (this participant was from the Replaced group).

4. Additional Learning Algorithm Performance Analysis

As discussed in the manuscript, the post-hoc nature by which this dataset may be analyzed provides a number of alternative opportunities to explore and hypotheses to be investigated. In an effort to provide the reader with additional information, such as performance benchmarks on varied feature sets and a deeper insight into the performance of the passive brain-computer interface (pBCI) system, additional learning trials have been constructed and executed to create the data tables, below. Each table was constructed with a specific feature set that was post-hoc simulated given consideration for the availability of features from unique data sources. Further, the learning algorithm performance (median from each of the k-folds, as described in the manuscript) is provided for each individual participant, separated by the between-subjects factors (electrode replacement).

4.1. All Features

The first feature set reported is that which contains all features described in the manuscript (EEG band power, V/HEOG band power, Blink Rate, and Inter-Beat Interval). All analysis presented in the manuscript used learning trials from this feature set. Results for this feature set are shown in Table 4.

Table 4 Learning algorithm performance results for the between-session test set using feature sets containing all available (37) features. Both accuracy (expressed as proportion of epochs correct, or '%') and sensitivity (expressed as d') are shown for each individual participant (each participant is individually identified by their 'pID'). Results in this table are the median values from the k-fold procedure performed for each participant and learning approach. Cross-session generalization is investigated by using either S1 or S2 as the learning set with S2 or S1 (respectively) reserved as the between-session test set.

	All Features (EEG Band Power, V/HEOG Band Power, Blink Rate, and Inter-Beat Interval)																
	pID	Train(S1)								Train(S2)							
		ANN		LDA		LIN-SVM		RBF-SVM		ANN		LDA		LIN-SVM		RBF-SVM	
		%	d'	%	d'	%	d'	%	d'	%	d'	%	d'	%	d'	%	d'
Electrodes Remained	05	.81	2.45	.86	2.13	.89	2.47	.78	1.75	.66	1.34	.67	1.14	.63	1.00	.67	1.21
	06	.68	2.43	.93	3.01	.88	2.98	.82	1.86	.88	2.50	.86	3.12	.87	2.24	.91	3.03
	08	.75	1.41	.80	1.67	.79	1.66	.78	1.58	.74	1.75	.72	1.74	.80	1.82	.75	1.75
	14	.81	2.19	.83	2.46	.77	2.20	.82	2.15	.87	2.27	.77	1.80	.80	1.78	.83	1.97
	16	.98	3.94	.97	3.80	.96	3.68	.87	3.45	.83	2.64	.87	2.87	.90	3.02	.66	2.40
	18	.83	1.95	.85	2.04	.84	1.99	.82	1.83	.80	1.81	.83	1.98	.81	1.85	.83	1.95
	23	.90	3.53	.89	3.38	.90	3.40	.90	3.05	.96	3.81	.96	3.56	.96	3.61	.92	3.90
	26	.95	3.41	.91	2.92	.86	2.74	.88	2.45	.96	3.65	.89	2.55	.90	3.01	.65	2.21
	28	.76	1.44	.82	1.88	.80	1.79	.66	.80	.84	2.12	.81	1.94	.84	1.99	.72	1.21
	31	.85	2.24	.89	2.56	.86	2.36	.79	1.99	.86	2.27	.82	2.47	.87	2.45	.81	1.82
Electrodes Replaced	01	.97	3.98	.91	2.86	.96	3.49	.92	3.47	.94	3.58	.94	3.42	.93	3.33	.93	3.02
	02	.74	1.92	.64	1.14	.74	1.88	.68	1.89	.74	1.35	.73	1.28	.83	2.02	.77	1.63
	04	.93	3.16	.93	2.89	.92	2.83	.93	3.31	.81	2.45	.78	2.39	.78	2.42	.87	2.55
	11	.65	.95	.79	1.81	.70	1.27	.66	1.00	.69	1.02	.84	2.37	.89	2.46	.60	.53
	15	.87	3.03	.88	3.74	.84	2.42	.90	3.55	.84	2.77	.86	2.95	.77	2.41	.82	2.66
	21	.99	4.96	.99	5.14	.98	4.67	.96	4.34	.99	4.48	.99	5.17	.98	4.17	.99	5.12
	24	.46	-.18	.42	-.43	.47	-.17	.45	-.26	.47	-.16	.45	-.25	.45	-.25	.41	-.44
	27	.61	.60	.70	1.08	.71	1.23	.59	.68	.63	.97	.73	1.43	.71	1.38	.56	.91
	29	.77	1.83	.86	2.24	.88	2.40	.62	1.34	.82	1.96	.82	1.85	.82	1.83	.72	1.42
	30	.86	2.88	.91	3.01	.86	2.87	.84	2.22	.92	3.10	.92	3.13	.93	3.05	.93	3.01

4.2. EEG Features

To examine the effectiveness of EEG-based features as compared to those derived from peripheral sources (ECG and V/HEOG), learning algorithm performance was evaluated using only the 25 EEG band power features. Results for this feature set are shown in Table 5.

Table 5 Learning algorithm performance results for the between-session test set using feature sets containing only the 25 EEG band power features. Data in this table was created from processes identical to and is formatted in the same structure as Table 4.

	EEG Features (EEG Band Power)																
	pID	Train(S1)								Train(S2)							
		ANN		LDA		LIN-SVM		RBF-SVM		ANN		LDA		LIN-SVM		RBF-SVM	
		%	d'	%	d'	%	d'	%	d'	%	d'	%	d'	%	d'	%	d'
E	05	.59	.49	.81	1.80	.80	1.79	.62	1.71	.84	3.50	.64	1.36	.62	1.12	.75	1.63

	06	.97	3.93	.90	3.25	.96	3.56	.96	3.58	.98	4.84	.85	3.57	.88	2.84	.92	3.10
	08	.75	1.36	.75	1.36	.75	1.35	.74	1.32	.90	3.23	.75	1.65	.76	1.60	.71	1.35
	14	.74	1.36	.77	1.69	.73	1.51	.65	1.26	.92	3.80	.73	1.52	.76	1.53	.67	.92
	16	.88	2.54	.91	2.72	.75	1.57	.81	2.45	.82	3.42	.83	2.45	.70	1.94	.67	2.00
	18	.83	1.90	.81	2.04	.80	1.94	.83	1.99	.95	3.53	.83	1.93	.82	1.88	.82	1.83
	23	.90	2.65	.92	2.93	.90	2.79	.88	2.35	1.00	5.77	.94	3.28	.95	3.27	.92	3.17
	26	.67	1.47	.67	1.61	.66	1.64	.72	1.28	.86	2.86	.64	.91	.61	.88	.76	1.46
	28	.72	1.20	.80	1.70	.80	1.67	.70	1.16	1.00	5.64	.82	1.99	.84	2.02	.71	1.12
	31	.71	1.62	.71	1.17	.76	1.46	.72	1.33	.52	.60	.79	2.04	.79	2.00	.79	2.11
Electrodes Replaced	01	.90	2.61	.85	2.28	.85	2.28	.79	2.01	.93	3.20	.94	3.31	.95	3.36	.88	2.49
	02	.58	.98	.56	.79	.55	.79	.56	1.17	.85	2.55	.64	.72	.74	1.31	.63	.73
	04	.84	2.04	.86	2.22	.86	2.17	.84	2.11	.98	4.68	.75	2.41	.78	2.08	.80	1.74
	11	.79	1.79	.80	1.87	.76	1.62	.76	1.54	.58	.98	.81	1.93	.85	2.10	.49	-.05
	15	.76	2.02	.82	3.41	.76	2.59	.76	2.59	.91	3.32	.80	2.61	.71	1.90	.88	2.36
	21	.98	4.73	.99	4.88	.98	4.77	.94	4.17	1.00	6.00	.99	5.02	.97	4.66	.92	4.08
	24	.49	-.07	.42	-.41	.44	-.32	.47	-.24	.83	2.07	.45	-.25	.44	-.32	.39	-.71
	27	.59	.46	.70	1.08	.70	1.11	.59	.50	.88	2.40	.69	.98	.67	.88	.57	.35
	29	.80	1.74	.82	1.86	.85	2.08	.77	1.52	.98	4.79	.72	1.25	.75	1.34	.75	1.39
	30	.84	2.01	.88	2.33	.84	2.03	.73	1.23	.82	3.23	.76	2.00	.76	1.96	.82	2.00

4.3. Peripheral Features

To examine the effectiveness of peripheral features (ECG and V/HEOG) as compared to those derived from EEG sources, learning algorithm performance was evaluated using only the 10 band power features from VEOG and HEOG as well as Blink Rate and Inter-Beat Interval. Results for this feature set are shown in Table 6.

Table 6 Learning algorithm performance results for the between-session test set using feature sets containing only the peripheral features (V/HEOG band power, Inter-Beat Interval, and Blink Rate). Data in this table was created from processes identical to and is formatted in the same structure as Table 4.

	Peripheral Features (V/HEOG Band Power, Blink Rate, and Inter-Beat Interval)																
	pID	Train(S1)								Train(S2)							
		ANN		LDA		LIN-SVM		RBF-SVM		ANN		LDA		LIN-SVM		RBF-SVM	
		%	d'	%	d'	%	d'	%	d'	%	d'	%	d'	%	d'	%	d'
Electrodes Remained	05	.58	.59	.80	1.79	.76	1.69	.50	.02	.57	.43	.94	3.18	.64	1.07	.50	.07
	06	.50	.16	.54	1.22	.50	.00	.51	.74	.68	1.14	.83	1.94	.77	1.62	.41	-.61
	08	.56	.36	.73	1.30	.74	1.36	.53	.19	.72	1.20	.84	1.99	.72	1.30	.74	1.32
	14	.76	1.75	.83	1.91	.82	1.96	.80	1.98	.78	1.59	.89	2.47	.81	1.77	.73	1.59
	16	.93	2.97	.98	4.30	.97	4.06	.88	2.38	.94	3.19	.98	4.18	.96	3.48	.91	2.70
	18	.60	.52	.64	.72	.64	.69	.62	.60	.56	.35	.68	.95	.65	.94	.54	.32
	23	.75	2.41	.72	2.30	.72	2.18	.72	1.58	.74	2.14	.87	2.30	.75	2.18	.72	2.00
	26	.96	3.52	.95	3.65	.97	3.77	.95	3.35	.98	4.27	.98	4.00	.98	4.44	.97	3.81
	28	.63	.72	.75	1.38	.70	1.11	.60	.52	.74	1.27	.87	2.23	.74	1.31	.66	.86
	31	.79	1.62	.90	2.79	.92	3.10	.80	1.77	.83	2.08	.97	3.72	.87	2.40	.83	1.98
Ele	01	.95	3.74	.97	4.69	.94	4.20	.91	3.44	.87	2.87	.98	4.33	.86	2.95	.88	2.66
	02	.67	.98	.68	1.06	.66	.96	.62	.69	.74	1.28	.86	2.13	.77	1.47	.73	1.23

	04	.91	2.72	.93	3.02	.96	4.02	.91	2.76	.84	2.54	.97	4.05	.89	2.85	.85	2.61
	11	.55	.40	.56	.42	.60	.67	.56	.37	.52	.12	.80	1.71	.58	.41	.55	.34
	15	.83	1.94	.87	2.30	.89	2.41	.81	1.80	.85	2.25	.93	2.91	.92	2.93	.83	1.98
	21	.72	1.17	.76	1.42	.75	1.33	.71	1.14	.84	2.03	.82	1.87	.82	1.86	.79	1.69
	24	.50	-.01	.48	-.08	.49	-.07	.48	-.14	.50	.01	.76	1.43	.54	.22	.51	.07
	27	.58	.43	.59	.46	.61	.61	.63	.68	.54	.38	.72	1.18	.58	.95	.53	.44
	29	.65	1.29	.88	2.35	.80	2.54	.53	.73	.73	1.32	.93	3.01	.78	1.81	.64	1.04
	30	.83	2.09	.87	2.79	.86	2.91	.83	2.00	.85	2.31	.93	3.01	.85	2.20	.86	2.51

4.4. EEG and Blink Rate Features

Since it is possible to derive a blink rate feature using EEG sources only, without the use of VEOG (e.g. Joyce, Gorodnitsky, and Kutas, 2004), the addition of Blink Rate to the EEG band power feature set was evaluated. Results for this feature set are shown in Table 7.

Table 7 Learning algorithm performance results for the between-session test set using feature sets containing EEG Band Power and Blink Rate features. Data in this table was created from processes identical to and is formatted in the same structure as Table 4.

	EEG Features (EEG Band Power) and Blink Rate																
	pID	Train(S1)								Train(S2)							
		ANN		LDA		LIN-SVM		RBF-SVM		ANN		LDA		LIN-SVM		RBF-SVM	
		%	d'	%	d'	%	d'	%	d'	%	d'	%	d'	%	d'	%	d'
Electrodes Remained	05	.65	.85	.86	2.17	.85	2.18	.61	1.87	.67	1.25	.66	1.27	.63	1.23	.79	1.64
	06	.92	2.88	.90	3.21	.91	2.85	.88	2.72	.86	2.39	.86	3.37	.83	1.94	.83	2.02
	08	.80	1.70	.79	1.63	.78	1.60	.78	1.58	.83	2.05	.82	2.34	.85	2.20	.80	1.88
	14	.77	1.62	.80	1.93	.75	1.69	.80	1.79	.79	1.66	.75	1.60	.78	1.65	.60	.86
	16	.95	3.51	.92	2.93	.88	2.41	.77	1.46	.71	2.05	.85	3.15	.74	2.21	.68	2.26
	18	.83	1.92	.83	2.04	.82	1.91	.84	1.96	.81	1.83	.83	1.92	.82	1.90	.81	1.82
	23	.90	3.37	.89	3.50	.89	3.25	.85	2.11	.96	3.70	.97	3.63	.96	3.63	.92	3.32
	26	.83	2.47	.73	2.06	.81	2.45	.84	2.11	.86	2.50	.81	2.04	.81	2.49	.84	2.47
	28	.76	1.50	.82	1.92	.80	1.73	.67	1.10	.83	1.95	.84	2.13	.86	2.16	.71	1.16
	31	.76	1.97	.87	2.24	.85	2.11	.74	1.66	.86	2.23	.83	2.35	.85	2.31	.82	2.01
Electrodes Replaced	01	.97	3.76	.91	2.88	.97	4.14	.85	2.93	.96	3.69	.94	3.35	.96	3.63	.97	3.80
	02	.63	1.31	.58	.94	.64	1.28	.60	1.43	.65	.82	.72	1.17	.74	1.34	.67	.95
	04	.92	2.93	.92	2.82	.90	2.60	.92	2.96	.91	2.93	.87	2.69	.91	2.98	.86	2.15
	11	.81	1.84	.86	2.27	.79	1.69	.77	1.50	.73	1.23	.84	2.17	.87	2.30	.48	-.08
	15	.85	2.52	.86	3.11	.83	2.34	.80	1.81	.83	2.49	.85	2.87	.78	2.27	.90	2.68
	21	.99	4.88	.99	5.02	.98	4.84	.92	3.96	.98	4.38	.99	5.07	.97	4.66	.92	4.06
	24	.47	-.17	.41	-.44	.44	-.34	.46	-.44	.41	-.44	.40	-.50	.41	-.47	.42	-.47
	27	.50	-.01	.62	.60	.63	.66	.53	.20	.64	.73	.69	.98	.67	.87	.59	.44
	29	.86	2.13	.87	2.25	.88	2.38	.84	2.03	.76	1.42	.74	1.36	.74	1.31	.76	1.44
	30	.88	2.44	.92	2.85	.90	2.66	.81	1.79	.91	3.75	.84	3.33	.88	3.14	.90	2.83

4.5. V/HEOG Features

Those features derived only from V/HEOG sources (V/HEOG Band Power and Blink rate) were evaluated for between-session learning algorithm performance. Results for this feature set are shown

in Table 8.

Table 8 Learning algorithm performance results for the between-session test set using feature sets containing only the features derived from V/HEOG sources (V/HEOG band power and Blink Rate). Data in this table was created from processes identical to and is formatted in the same structure as Table 4.

	V/HEOG Features (V/HEOG Band Power and Blink Rate)																
		Train(S1)								Train(S2)							
	pID	ANN		LDA		LIN-SVM		RBF-SVM		ANN		LDA		LIN-SVM		RBF-SVM	
		%	d'	%	d'	%	d'	%	d'	%	d'	%	d'	%	d'	%	d'
Electrodes Remained	05	.65	.80	.80	1.65	.79	1.62	.57	.39	.62	.70	.67	.96	.67	1.11	.64	.91
	06	.64	.86	.65	1.14	.66	1.13	.64	.84	.70	1.09	.73	1.21	.71	1.13	.58	.45
	08	.70	1.13	.71	1.30	.73	1.33	.71	1.17	.74	1.31	.75	1.37	.76	1.44	.75	1.36
	14	.81	1.80	.80	1.72	.81	1.78	.78	1.60	.75	1.40	.77	1.64	.76	1.58	.77	1.47
	16	.93	2.94	.97	3.85	.92	2.91	.92	2.84	.92	2.88	.93	2.89	.94	3.05	.90	2.60
	18	.57	.39	.64	.73	.64	.70	.61	.57	.60	.54	.74	1.46	.75	1.35	.55	.27
	23	.71	2.45	.71	2.66	.72	2.90	.68	1.22	.72	1.98	.76	2.31	.75	2.19	.72	2.19
	26	.88	2.66	.91	2.87	.91	3.08	.90	2.71	.92	3.10	.91	3.05	.93	3.34	.91	3.06
	28	.65	.87	.73	1.36	.72	1.27	.62	.73	.68	.96	.73	1.26	.73	1.24	.65	.78
	31	.85	2.11	.90	2.79	.93	3.18	.83	1.90	.83	1.96	.86	2.30	.87	2.27	.80	1.82
Electrodes Replaced	01	.95	3.60	.98	4.23	.97	4.56	.96	3.43	.94	3.17	.93	3.25	.93	3.14	.91	2.72
	02	.63	.87	.67	1.02	.66	.94	.59	.60	.77	1.50	.78	1.58	.77	1.52	.72	1.26
	04	.93	3.04	.95	3.43	.96	4.31	.94	3.27	.91	2.97	.83	2.45	.91	3.07	.90	2.88
	11	.50	.01	.56	.36	.60	.60	.50	.00	.54	.29	.55	.36	.57	.49	.53	.26
	15	.82	1.92	.88	2.31	.89	2.48	.82	1.85	.85	2.36	.91	2.98	.91	2.94	.81	2.06
	21	.74	1.28	.77	1.48	.75	1.38	.72	1.19	.80	1.74	.86	2.14	.82	1.84	.71	1.33
	24	.54	.19	.49	-.05	.49	-.03	.50	.00	.50	-.01	.49	-.08	.53	.16	.50	.01
	27	.62	.67	.60	.51	.61	.60	.61	.63	.60	.54	.66	1.09	.63	.93	.59	.52
	29	.86	2.27	.86	2.45	.88	2.58	.83	1.96	.79	1.65	.82	1.88	.82	1.93	.77	1.53
	30	.83	2.13	.87	2.83	.87	2.93	.81	1.95	.86	2.38	.84	2.18	.85	2.18	.87	2.68

4.6. Features with Optical Measurement Methods (Inter-Beat Interval and Blink Rate)

Two of the peripheral features, Inter-Beat Interval and Blink Rate, are feasible to measure via remote, optical methods using imaging approaches. For example, Zhu and Ji (2007) describe a remote, imaging-based eye tracking system, and Estepp, Blackford, and Meier (2014) describe an imaging-based pulse rate measurement system. Given the uniqueness of being able to measure these features without physical contact with the participant, they were evaluated in tandem. Results for this feature set are shown in Table 9.

Table 9 Learning algorithm performance results for the between-session test set using feature sets containing only two peripheral features (Inter-Beat Interval and Blink Rate). Data in this table was created from processes identical to and is formatted in the same structure as Table 4.

Features with Optical Measurement Methods (Inter-Beat Interval and Blink Rate)																
	Train(S1)								Train(S2)							
pID	ANN		LDA		LIN-SVM		RBF-SVM		ANN		LDA		LIN-SVM		RBF-SVM	
	%	d'	%	d'	%	d'	%	d'	%	d'	%	d'	%	d'	%	d'

Electrodes Remained	05	.45	.01	.60	1.43	.55	1.08	.59	1.57	.51	.20	.56	.50	.53	.32	.50	.16
	06	.50	.00	.49	-.80	.50	.00	.50	-.21	.51	.42	.56	1.44	.50	.31	.29	-2.8
	08	.70	1.35	.76	1.41	.74	1.37	.63	.97	.74	1.70	.79	1.96	.69	1.99	.74	1.30
	14	.67	1.94	.76	2.07	.71	2.08	.67	2.09	.74	1.80	.71	1.16	.71	1.73	.65	1.71
	16	.91	2.71	.92	2.78	.90	2.64	.76	1.47	.89	2.69	.89	2.78	.92	2.85	.82	1.83
	18	.49	-.05	.53	.14	.52	.10	.47	-.13	.48	-.15	.52	.19	.50	-.06	.48	-.20
	23	.63	2.38	.68	2.21	.74	1.56	.63	.88	.62	1.45	.62	1.59	.62	1.74	.69	1.62
	26	.92	3.16	.93	3.89	.93	3.73	.73	1.55	.97	3.88	.97	3.95	.97	3.95	.91	3.13
	28	.59	.73	.63	.85	.60	.74	.61	.58	.73	1.23	.64	.71	.66	.81	.67	.96
	31	.87	2.49	.90	2.58	.90	2.58	.74	1.42	.69	1.48	.77	1.79	.75	1.72	.65	1.20
Electrodes Replaced	01	.88	3.30	.91	3.30	.83	3.47	.72	1.82	.72	2.16	.84	2.60	.72	2.12	.75	2.40
	02	.76	1.70	.82	1.93	.78	1.85	.69	.98	.65	.78	.64	.72	.63	.79	.67	.90
	04	.94	3.46	.94	3.28	.93	3.75	.93	3.39	.94	3.19	.98	4.12	.93	3.07	.92	2.90
	11	.52	.36	.53	.23	.52	.19	.51	.05	.51	.26	.52	.31	.51	.21	.49	-.12
	15	.82	1.84	.84	2.01	.83	1.94	.81	1.79	.83	2.08	.84	2.10	.83	2.01	.83	1.92
	21	.70	1.02	.69	1.03	.70	1.06	.68	1.03	.89	2.65	.88	2.40	.88	2.32	.75	1.33
	24	.55	.31	.43	-.34	.42	-.54	.49	-.05	.53	.18	.56	.35	.53	.19	.48	-.09
	27	.50	.01	.49	-.04	.50	-.02	.51	.03	.48	-.17	.49	-.14	.49	-.17	.47	-.27
	29	.51	.50	.75	1.47	.56	.93	.50	.05	.51	.11	.64	.87	.65	.77	.69	1.00
	30	.82	2.16	.81	1.98	.82	2.35	.83	1.90	.91	2.85	.95	3.43	.75	2.26	.88	2.63

4.7. Wearable Sensor Technology and Inter-Beat Interval

As a feature that is readily obtainable from wearable sensor technology (e.g. Pantelopoulos and Bourbakis, 2010), Inter-Beat Interval as a single-feature approach was evaluated. Results for this feature set are shown in Table 10.

Table 10 Learning algorithm performance results for the between-session test set using feature sets containing only Inter-Beat Interval. Data in this table was created from processes identical to and is formatted in the same structure as Table 4.

		Single Feature (Inter-Beat Interval)															
	pID	Train(S1)								Train(S2)							
		ANN		LDA		LIN-SVM		RBF-SVM		ANN		LDA		LIN-SVM		RBF-SVM	
		%	d'	%	d'	%	d'	%	d'	%	d'	%	d'	%	d'	%	d'
Electrodes Remained	05	.52	.72	.50	.21	.51	.33	.52	.96	.51	.68	.51	1.00	.51	1.09	.51	.64
	06	.50	.00	.50	.21	.50	.00	.50	.00	.53	.91	.52	.80	.52	.76	.46	-.07
	08	.50	.00	.49	-.26	.48	-.28	.56	.33	.50	.00	.50	.05	.50	.10	.50	.12
	14	.59	1.43	.61	1.56	.61	1.57	.61	1.48	.60	1.34	.57	1.28	.59	1.32	.59	1.57
	16	.61	.72	.59	.72	.60	.76	.60	.67	.57	1.44	.64	1.83	.64	1.83	.57	1.79
	18	.49	-.34	.49	-.60	.41	-.74	.41	-.63	.44	-.51	.45	-.55	.44	-.56	.44	-.45
	23	.52	.13	.53	.18	.53	.16	.54	.20	.59	.86	.65	.88	.65	.90	.51	.07
	26	.88	2.78	.87	2.84	.88	2.78	.66	.88	.87	2.27	.87	2.26	.85	2.17	.83	1.96
	28	.54	.34	.53	.20	.53	.23	.54	.32	.65	1.37	.65	1.73	.55	1.24	.64	.72
	31	.50	.00	.49	-.47	.49	-.14	.48	-.08	.49	-.31	.49	-.29	.49	-.27	.50	.02
Electr	01	.60	2.16	.59	2.15	.59	2.15	.60	2.07	.55	1.27	.55	1.24	.55	1.31	.54	1.30
	02	.56	.58	.59	.53	.58	.52	.56	.56	.52	.20	.53	.22	.52	.17	.52	.16
	04	.80	1.78	.73	1.32	.76	1.45	.76	1.44	.59	.63	.59	.62	.59	.60	.60	.64

	11	.50	.00	.50	-.44	.48	-.46	.58	.44	.51	.24	.51	.26	.51	.26	.52	.20
	15	.59	.85	.62	.71	.62	.73	.51	.06	.55	.27	.48	-.10	.50	-.03	.53	.16
	21	.62	.64	.62	.59	.62	.62	.60	.54	.72	1.31	.73	1.31	.74	1.31	.67	.94
	24	.56	.47	.58	.46	.57	.50	.45	-.26	.47	-.23	.48	-.09	.49	-.07	.46	-.31
	27	.50	.00	.49	-.29	.50	.00	.41	-.77	.48	-.23	.48	-.33	.48	-.28	.48	-.17
	29	.48	-1.2	.48	-1.2	.48	-1.2	.48	-1.1	.48	-.63	.49	-.32	.48	-.60	.48	-1.0
	30	.60	.58	.60	.58	.60	.60	.59	.55	.58	.78	.61	.80	.60	.82	.56	.42

5. References

Comstock, J. R., and Arnegard, R. J., (1992). The multi-attribute task battery for human operator workload and strategic behavior research. NASA Technical Memorandum No. 104174.

Estepp, J. R., Blackford, E. B., and Meier, C. M. (2014). Recovering pulse rate during motion artifact with a multi-imager array for non-contact imaging photoplethysmography. *Proceedings of the 2014 IEEE International Conference on Systems, Man, and Cybernetics*, 1481-1488.

Joyce, C. A., Gorodnitsky, I. F., and Kutas., M. (2004). Automatic removal of eye movement and blink artifacts from EEG data using blind component analysis separation. *Psychophysiology*, *41*(2), 313-325.

Miller, W. D. (2010). The U.S. Air Force-developed adaptation of the Multi-Attribute Task Battery for the assessment of human operator workload and strategic behavior (Technical Report AFRL-RH-WP-TR-2010-0133). Retrieved from the Defense Technical Information Center website: http://www.dtic.mil/dtic/tr/fulltext/u2/a537547.pdf

Pantelopoulos, A., and Bourbakis, N. G. (2010). A survey on wearable senor-based systems for health monitoring and prognosis. *IEEE Transactions on Systems, Man, and Cybernetics, Part C: Applications and Reviews*, *40*(1), 1-12.

Zhu, Z., and Ji, Q. (2007). Novel eye gaze tracking techniques under natural movement. *IEEE Transactions on Biomedical Engineering*, *54*(12), 2246-2260.

Wilson, G. F., and Russell, C. A. (2007). Performance enhancement in an uninhabited air vehicle task using psychophysiologically determined adaptive aiding. *Human Factors*, *43*, 1005–1018.
